# AI-based staging, causal hypothesis and progression of subjects at risk of Alzheimer’s disease: a multicenter study

**DOI:** 10.3389/fneur.2025.1568086

**Published:** 2025-05-19

**Authors:** Simona Aresta, Raffaello Nemni, Moreno Zanardo, Graziella Sirabian, Dario Capelli, Marco Alì, Paolo Vitali, Enrico Giuseppe Bertoldo, Valentina Fiolo, Lilla Bonanno, Giuseppa Maresca, Petronilla Battista, Francesco Sardanelli, Francesca Benedetta Pizzini, Isabella Castiglioni, Christian Salvatore

**Affiliations:** ^1^Department of Science, Technology and Society, University School for Advanced Studies IUSS Pavia, Pavia, Italy; ^2^Centro Diagnostico Italiano S.p.A., Milan, Italy; ^3^Unit of Radiology, IRCCS Policlinico San Donato, San Donato Milanese, Italy; ^4^Department of Biomedical Sciences for Health, Università degli Studi di Milano, Milan, Italy; ^5^Clinical Psychology Service, IRCCS Policlinico San Donato, San Donato Milanese, Italy; ^6^IRCCS Centro Neurolesi Bonino Pulejo, Messina, Italy; ^7^Istituti Clinici Scientifici Maugeri IRCCS, Laboratory of Neuropsychology, Institute of Bari, Bari, Italy; ^8^Lega Italiana per la Lotta contro i Tumori (LILT) Milano Monza Brianza, Milan, Italy; ^9^Department of Engineering for Innovation Medicine, University of Verona, Verona, Italy; ^10^Department of Physics “G. Occhialini”, Università degli Studi di Milano-Bicocca, Milan, Italy; ^11^Deeptrace Technologies S.R.L., Milan, Italy

**Keywords:** Alzheimer’s disease, artificial intelligence, MRI, neuropsychological scores, staging, diagnosis

## Abstract

**Introduction:**

In 2024, 11 European scientific societies/organizations and one patient advocacy association have defined a patient-centered biomarker-based diagnostic workflow for memory clinics evaluating neurocognitive disorders.

**Methods:**

We tested the performance of an artificial intelligence (AI) tool applied to neuropsychological and magnetic resonance imaging (MRI) assessment for staging and causal hypothesis, which are the two recommended workflow steps guiding the next one recommending optimal biomarkers to be used for a biological diagnosis of neurocognitive disorders, according to intersocietal recommendations. Moreover, we assessed the AI performance in predicting the progression to Alzheimer’s disease (AD)-dementia.

**Results:**

For the three-class classification of staging (n patients = 426), the inter-rater AI-humans agreement was substantial for both healthy subjects/subjective cognitive impairment/worried-well vs. all the remaining groups (rest) (Cohen’s *κ* = 0.81) and mild cognitive impairment/mild dementia vs. rest *κ* = 0.70) classification, almost perfect for moderate/severe dementia vs. rest *κ* =0.90) classification. For the three-class classification of causal hypotheses (*n* = 112), the AI performance vs. biomarker-based diagnosis was: positive predictive value 91% [95% CI: 84–96%]; negative predictive value 100%, and accuracy 91% [84–96%]. For the binary classification of progression or not progression to AD-dementia at 24-month, with clinical conversion as a reference standard (*n* = 341), the AI performance was: sensitivity 89% [84–94%], specificity 82% [77–87%]; accuracy 85% [81–89%]; and area under the receiver operating characteristic curve 83% [79–87%].

**Discussion:**

The AI tool showed high agreement with human assessment for staging, high accuracy with biomarkers for causal hypotheses of neurocognitive disorders and predicted progression to AD at 24-month with 89% sensitivity and 82% specificity.

## 1 Introduction

Alzheimer’s disease (AD) is the most prevalent neurodegenerative disorder globally, caused by the accumulation of beta-amyloid protein and the development of neurofibrillary tangles that can lead over time to a severe form of dementia (1). It accounts for 60–70% of all dementia cases worldwide, with over 50 million individuals currently affected and nearly 10 million new cases diagnosed each year (2). The prevalence increases with age, with one new case occurring approximately every 3 s globally (3), and in the future, it is expected to increase in parallel with the aging of the population (4). The burden of AD intensifies as the condition progresses, encompassing not only direct medical expenses but also impacting caregivers and families, long-term health systems sustainability, economies, and society at large (5).

The literature agrees on the need to identify early stages of the disease to anticipate already known diagnostic protocols, as well as to allow a more efficient selection of subjects who could benefit from new disease-modifying therapies (6). The current scarcity of therapies may block or slow the progression to AD-dementia due to a low ability to select the appropriate population, taking also into account that the effectiveness of the treatment may increase with its anticipation.

The diagnosis of AD is based on biological tests following lumbar puncture and measurement of cerebrospinal fluid (CSF) biomarkers: phosphorylated-tau (p-tau) or total-tau (t-tau), amyloid-β42 (Aβ42), and amyloid-β42-to-amyloid-β40 ratio (Aβ42/Aβ40). Revised diagnostic criteria for AD introduced in 2011 emphasized the use of medical imaging to identify objective signs of the disease in the brain, such as amyloid-beta (Aβ) or tau-specific positron emission tomography (PET) imaging (7, 8). PET studies provide high specificity but are quite expensive, invasive (due to exposure to ionizing radiation), and with limited access for patients, particularly in low- and middle-income countries (9). In contrast, magnetic resonance imaging (MRI) is more widely available, noninvasive, and cost-effective, making it a valuable tool for detecting AD-related neurodegeneration and monitoring disease progression and prognosis (10, 11).

However, the choice of a patient’s workflow and tests for biomarkers is often defined by organizational and logistical factors rather than by clinical factors and patient preferences. Currently, the clinical diagnosis of AD primarily relies on the self-reported cognitive complaints (or those reported by caregivers) as well as clinicians’ observations of cognitive, functional, and behavioral symptoms throughout the disease progression (12–14).

Delegates from 11 European scientific societies and organizations and a patient advocacy association (Alzheimer Europe), have recently defined a patient-centered, biomarker-based diagnostic workflow to be used in specialized clinical contests, in particular in memory clinics (15). Common practices in memory clinics guided the workflow (16, 17). The first wave (wave 0) is a clinical examination and assessment of the subject’s complaints, aimed at excluding secondary causes for the cognitive complaint and staging patients as having mild cognitive impairment (MCI) or mild dementia (MD) in order to undergo the following steps for a biomarker-based diagnosis. Individuals with moderate-to-severe dementia as well as subjective cognitive impairment (SCI) or worried well (WW) subjects are also important to be staged but they would not typically proceed in the workflow being generally not considered appropriate for a biomarker-based diagnosis. History, physical and neurological examinations, cognitive screening tests and functional assessment can be used for this first purpose in wave 0. Patients are then categorized into clinical syndromes, according to the patient’s salient clinical, cognitive and structural neuroimaging findings. The clinical syndrome allows clinical diagnosis based on hypotheses of disease causation that direct the selection of first-line biomarkers. According to the results of first-line biomarkers, other second-line biomarkers might be measured. Considering AD, the diagnostic process is conclusive for AD cause when CSF biomarkers indicate brain amyloidosis and tau pathology (based on reduced CSF Aβ42 or Aβ42/Aβ40 ratio and elevated p-tau protein) (18).

Currently, the prodromal stage of AD-dementia is considered to be amnestic MCI (aMCI), a syndrome that causes objectifiable alterations mainly affecting the cognitive domain of memory, without satisfying the criteria for the diagnosis of dementia and therefore placing itself between the cognitive decline caused by normal aging and dementia itself (19, 20). The overall prevalence of aMCI in population epidemiological studies varies between 3 and 19% in the population over 65 years of age (21). Although the general tendency of subjects with aMCI is progression to AD-dementia, some subjects evolve faster than others; for this reason some authors have differentiated aMCI depending on whether or not they show an evolution to AD-dementia after 24-month from the first diagnosis of aMCI (22).

Regarding patient’s clinical and cognitive findings, although no standard neuropsychological battery tests have been defined around the world, experts agree that a detailed neuropsychological assessment should include tests assessing memory and learning, working memory, language, visuoconstructional reasoning, complex attention and functional abilities (23).

Regarding patient’s structural neuroimaging findings, manual segmentation of MRI images requires long times, limits reproducibility and does not allow for the best evaluation of the atrophy, also because some volumetric variations associated with the evolution into AD are not recognizable when viewed by human readers, particularly in early stages (24). To overcome these difficulties, MRI analysis methods are being spread mainly based on supervised machine learning techniques, i.e., on algorithms that automate classification and prediction tasks (12, 25).

**Figure 1:**
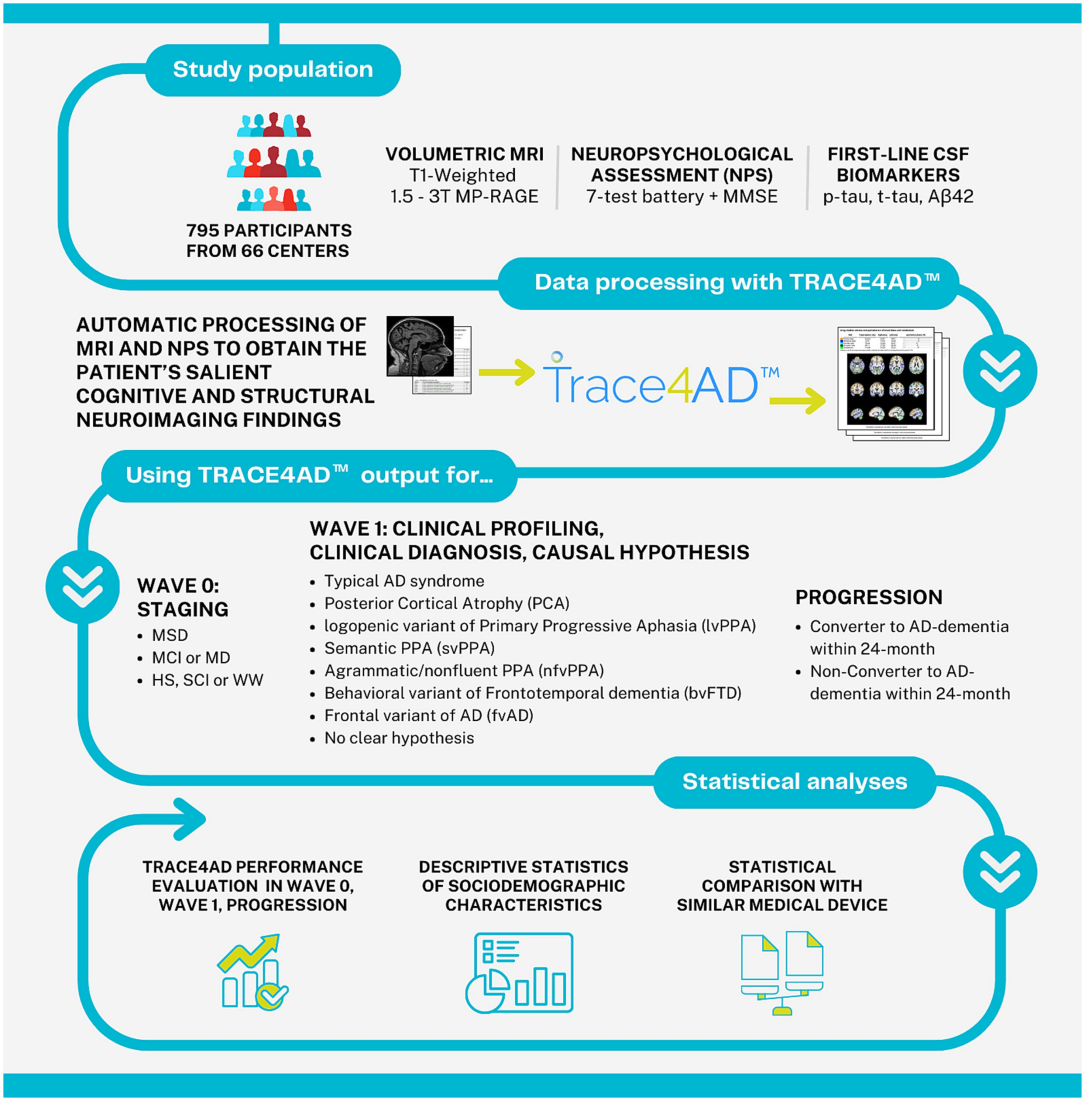
Graphical representation of the study pipeline. MRI, magnetic resonance imaging; MP-RAGE, magnetization prepared rapid gradient echo imaging; NPS, neuropsychological assessment; MMSE, mini-mental state examination; CSF, cerebrospinal fluid; A*β*42, amyloid-β protein 42; MSD, moderate-to-severe dementia; MCI, mild cognitive impairment; MD, mild dementia; HS, healthy subjects; SCI, subjective cognitive decline; WW, worried well.

The aim of this study was to evaluate the clinical performance of an AI tool applied to neuropsychological assessment and MRI for supporting the staging, clinical profiling, diagnosis, causal hypothesis, and progression of subjects at risk of AD following the above-mentioned intersocietal recommendations. A graphical representation of the study pipeline is reported in Figure 1.

## 2 Materials and methods

### 2.1 Study population

This observational, multicentric study included subjects clinically examined and assessed, excluding secondary causes for the cognitive complaint, and staged as healthy subjects (HS), with subjective cognitive impairment (SCI), MCI, or AD-dementia at baseline and at 24-month follow-up. Patients were clinically profiled into AD clinical syndrome by summarizing the patient’s salient clinical/cognitive characteristics and structural neuroimaging findings. First-line biomarkers p-tau, t-tau, and Aβ42 were measured.

Patients were enrolled from 63 centers of the Alzheimer’s Disease Neuroimaging Initiative (ADNI) (US/Canada), (26), following the ADNI retrospective clinical protocol, from two Italian centers, Centro Diagnostico Italiano (Memory Clinic-CDI, Milan, Italy), IRCCS Policlinico San Donato-Università degli Studi di Milano, Milan, Italy (San Donato Milanese, Milan, Italy), following the retrospective and prospective clinical protocol “White Matter Hyperintensity” (WMH-AD, NCT06179680; date of approval: 8-June 2022), and from another Italian center, IRCCS Centro Neurolesi Bonino Pulejo (BP, Messina, Italy), following the retrospective clinical protocol (protocol code: 08/2022, date of approval: 21 July 2022). All the patients signed an informed consent to participate in the studies.

ADNI was launched in 2003 as a public-private partnership, led by Principal Investigator Michael W. Weiner, MD, and supported by the National Institute on Aging, the foundation for the National Institutes of Health, the Alzheimer’s associations, and dozens of companies. The primary goal of ADNI has been to test whether serial MRI, PET, other biological markers, and clinical and neuropsychological assessment can be combined to measure the progression of MCI and early AD (26). For up-to-date information, see www.adni-info.org. The ADNI enrollment process is subdivided in different phases: ADNI 1 (2004–2009), ADNI GO (2009–2011), ADNI 2 (2010–2017), ADNI 3 (2017–2022), and ADNI 4 (2022–current). Inclusion criteria for all the subjects subgroups are: (1) age between 55 and 90 years old, (2) a study partner able to provide independent functioning evaluation, (3) English or Spanish native speakers, (4) willingness to participate, and (5) ability to perform different tests, neuroimaging, at least one lumbar puncture and all follow-up visits [further information can be retrieved here (27)]. The inclusion criteria for HS were: Mini-Mental State Examination (MMSE) (28) between 24 and 30, Clinical Dementia Rating (CDR) (29) equal to zero, normal memory function documented by scoring at specific cutoffs on the Logical Memory II subscale from Wechsler Memory Scale (30), without significant impairment in cognitive functions or activity of daily living, absence of dementia, and Geriatric Depression Scale (GDS) score (31) minor than 6. The inclusion criteria for MCI were: MMSE between 24 and 30, CDR equal to 0.5, memory complaints by the subject or study partner, abnormal memory function documented by scoring below the education-adjusted cutoff on the Logical memory II subscale from the Wechsler Memory Scale-Revised, general cognition and functional performance sufficiently preserved, and GDS score minor than 6. The inclusion criteria for AD-dementia clinical syndrome were: MMSE between 20 and 26, CDR equal to 0.5 or 1.0, memory complaints by the subject or study partner, abnormal memory function documented by scoring below the education-adjusted cutoff on the Logical memory II subscale from the Wechsler Memory Scale-Revised, criteria for probable AD as defined by the National Institute of Neurological and Communicative Disorders and Stroke (NINCDS) and by the Alzheimer’s Disease and Related Disorders Association (ADRDA) (32, 33), and GDS score minor than 6.

The WMH-AD study was started in 2022 with the primary goal of measuring the extent and distribution of white matter hyperintensities in the brains of individuals with aMCI or a clinical diagnosis of AD. It is an observational clinical study, which included age- and sex-matched subjects without cognitive impairment or significant neurological disorders. Subjects underwent neurological, neuropsychological assessments, and neuroimaging procedures. The inclusion criteria for all the subject subgroups from CDI and Policlinico San Donato-Università degli Studi di Milano, Milan, Italy were age greater than or equal to 45 years old, The inclusion criteria for AD subjects: (1) meeting established criteria for AD diagnosis, including cognitive and memory deficits along with functional impairment, (2) without comorbidities that could affect cognitive function, and (3) without other forms of dementia. In particular, inclusion criteria for aMCI subjects: (1) subjects diagnosed with amnestic-MCI; (2) not meeting criteria for an AD diagnosis, and (3) absence of any form of neurological condition that might mimic or contribute to cognitive impairment. Inclusion criteria for HS: (1) normal cognitive function for their age; (2) absence of memory complaints; and (3) no history of neurological or psychiatric disorders.

### 2.2 Clinical data collection

#### 2.2.1 Neuropsychological assessment

The patient’s salient cognitive characteristics were obtained by a detailed neuropsychological battery, including eight neuropsychological tests assessing different cognitive domains. Global cognitive efficiency was tested using Mini-Mental State Examination (MMSE) (28), auditory verbal memory and verbal learning by AVLT (immediate, delayed, and recognition) (34), attention and executive functions by Symbol Digit (35), Trail Making Test (TMT-A, TMT-B) (36), and Digit Span (Forward and Backward) (37), visuo-constructive abilities by Clock (38), language by Category Fluency Test (animals-vegetables) (39), and the Boston Naming Test (BNT) (40). Functional Assessment Questionnaire (FAQ) was used to assess functional activities of daily living (41).

#### 2.2.2 MRI studies

The subject’s structural neuroimaging findings were obtained from brain MRI studies. The acquisition protocol was designed to focus on brain morphometry, always utilizing a T1-weighted 3D volumetric imaging method through the Magnetization Prepared RApid Gradient Echo (MP-RAGE) protocol. The protocol began with a scout scan to achieve anatomical orientation in sagittal, coronal, and transverse planes. Following the scout scan, the main MP-RAGE scan was performed. This scan ensures the complete inclusion of the skull superiorly and laterally, as well as the cerebellum inferiorly, and incorporates the nose in the anterior–posterior plane to prevent missing details that could affect data processing. Images were then reconstructed with isotropic voxel dimensions of approximately 1 mm^3^, with a maximum of 1.5 mm in any direction to eliminate directional bias and maintain high spatial resolution.

#### 2.2.3 First-line biomarkers

CSF biomarkers were measured: p-tau, t-tau, and Aβ42. CSF was collected through a lumbar puncture using a small-caliber atraumatic needle, such as a 24- or 25-gauge Sprotte needle. To remove any blood from minor trauma caused during needle insertion, the first 1–2 mL of CSF (or more if necessary) were discarded. Following this, 20 mL of CSF were collected for analysis and processing: (1) initial testing (the first 3 mL of CSF were used for standard laboratory tests, including cell counts, glucose, and total protein, conducted at local laboratories); (2) further processing (the remaining CSF was collected and processed). All collected samples were placed in containers with dry ice, except samples designated for immortalized cell lines and ApoE genotyping, which were shipped at room temperature. Samples were dispatched the same day via express mail with overnight delivery to the Penn AD Biomarker Fluid Bank Laboratory. Upon receipt at the laboratory, samples were thawed, aliquoted into labeled plastic vials, and stored in designated −80°C freezers. The samples were inventoried and tracked using specialized software. A barcoding system ensured accurate tracking and data management.

Aβ42, t-tau, and p-tau were analyzed using Elecsys amyloid-β42 CSF, Elecsys total-tau CSF, and Elecsys phosphorylated-tau (181P) CSF electrochemiluminescence immunoassays (Roche Diagnostics International Ltd., Rotkreuz, Switzerland) (42–44).

2-[^18^F]fluoro-2-deoxy-D-glucose ([^18^F]FDG) PET biomarker was also measured giving information on patterns of cortical hypometabolism that are indicative of neurodegenerative diseases (e.g., Alzheimer’s disease, frontotemporal dementia, Lewy body disease, motor tauopathies).

[^18^F]FDG PET was reported as positive or negative based on the abnormal results specific for the clinical syndrome, i.e., the hypometabolic pattern involving the posterior cingulate cortex, precuneus, posterior temporoparietal cortex, and medial temporal lobe for AD; the hypometabolism of the frontal or anterior temporal regions for bvFTD; the hypometabolism pattern of the left posterior fronto-insular cortex for non-fluent PPA; and the hypometabolism of the anterior temporal regions for semantic PPA, according to European Intersocietal recommendation (15).

The diagnostic process was conclusive for AD cause when CSF biomarkers indicated brain amyloidosis (based on reduction of CSF Aβ42 or Aβ42/Aβ40 ratio) and tau pathology (based on elevated p-tau protein), based on the ratio t-tau and Aβ42 major than 0.23 (biological diagnosis) (45).

In case of Aβ42+ t-tau- or Aβ42− t-tau+, the diagnosis was concluded when [^18^F]FDG PET gave abnormal results specific for the clinical syndrome according to European Intersocietal recommendation (15).

### 2.3 Data processing

The AI-based software TRACE4AD™ (DeepTrace Technologies, Milan, Italy) (12) was used to automatically process the MRI brain study and the neuropsychological tests of each subject in order to obtain the patient’s salient cognitive and structural neuroimaging findings.

The software is a CE-marked medical device intended for use by neurologists, neuropsychologists and neuroradiologists, supporting them for staging, clinical profiling, clinical diagnosis and prognosis of subjects at risk of AD, leaving ultimate decision-making to the clinicians for the patient biomarker’s based diagnosis, clinical diagnosis and management. Details on the TRACE4AD software can be found in (12). TRACE4AD is a cloud-based solution offering a full PACS integration and also being compliant with standard data formats for both MRI images and clinical reports. A memory clinic can adopt the tool by uploading the MRI study of the patient or by automatically receiving the MRI study, when the PACS integration is preferred. Scores of neuropsychological tests can be uploaded in the software in standard formats. The manufacturer (DeepTrace Technologies Srl) is ISO 13485 certified. TRACE4AD was developed in accordance with the latest and highest standards of safety and security for AI-based medical devices, including BS AAMI 34971:2023 (46), IEC 81001–5-1:2021 (47), MDCG 2019-16 (48), as well as with European Regulation 2024/1689 (AI ACT) (49), European Regulations 2016/679 (50), 2018/1725 (51) and European Directive 2016/680 (52). TRACE4AD allows remotely controlled updates. Customer support is provided. Clinicians are provided with training material and live demonstrations. The tool offers an operating manual and other guidance documentation, including MRI and neuropsychological testing protocols and data for using the tool. An online remote training is provided by the product-specialist team before starting to use the tool with verification of effectiveness. We summarize herein the main steps of the software workflow.

For each subject, the software performs an automatic segmentation of the T1-weighted 3D brain MRI study in order to extract brain-volumetric features for atrophy assessment (in particular regarding the gray matter). Image pre-processing includes: (1) image re-orientation, (2) cropping, (3) skull-stripping, and (4) image normalization to the Montreal Neurological Image (MNI) standard space by means of coregistration of brain volume to the MNI template (MNI152 T1 1 mm brain) (53, 54). A voxel-based statistical inference method was used by an automatic AI classifier to identify areas of atrophy due to neuronal death, in particular in the entorhinal cortex, which is one of the first regions of the hippocampus to atrophy in the early stages of AD, or in the mid-temporal cortex and the temporal pole. These are biomarkers of clinical progression and evolution consistent with the pathological studies by Braak et al. (14), demonstrating that during the development of AD pathology, tau protein tangles increase, associated with synapse loss and neurodegeneration. The architecture of the AI classifier is based on an ensemble of Support Vector Machines (SVMs) with a classification voting scheme based on the ensemble consensus. The feature extraction and selection method is based on Principal Component Analysis (PCA) coupled to Fisher Discriminant Ratio (FDR). For each study, the software also performed an assessment and extracted cognitive features from a detailed battery of neuropsychological tests assessing memory and learning, attention and executive function, visuospatial ability, language and fluency, and functional activities. The Italian version of the neuropsychological tests used in TRACE4AD has been psycho-linguistically adapted and made comparable to the American neuropsychological battery used in ADNI. Cognitive features were combined with atrophy features to automatically classify the subject in different classes. As final output, for each subject, the software provides a report with the cognitive deficits, the measured brain-volumetric features and the predicted individual risk of conversion to AD-dementia within the following 24-month (low risk, LR; high risk, HR), supporting neurologists, neuropsychologists and neuroradiologists in staging, clinical profiling, diagnosis, prognosis, and decision-making.

#### 2.3.1 Subgroup analysis 0: staging

The tool was used to stage subjects in the following distinct classes:

1) “Moderate-to-severe dementia (MSD)” was classified when, in the tool report, either three functional impairment or at least three cognitive impairments were reported, and MMSE ≤ 26;
2) MCI or MD were classified when, in the tool report, one or more cognitive impairments and no significant functional impairments were reported, and MMSE > 26;
3) “HS, SCI or WW” were classified when, in the tool report, no memory impairment or no significant impairment in cognitive functions or activity of daily living was reported, and MMSE ≥ 24.

The cognitive features automatically processed by the AI tool were used to detect cognitive impairments in specific domains when compared with normative cut-offs (55–59).

The clinical performance of the AI tool in classifying subjects as HS/SCI/WW, MCI/MD, and MSD was evaluated with respect to clinical staging performed by clinicians at baseline and at 24-month follow-up in terms of percentage agreement for each stage (Performance 0 staging: AI tool vs. clinicians).

#### 2.3.2 Subgroup analysis I: clinical profiling, clinical diagnosis and causal hypothesis

The tool was used to profile subjects in the following distinct classes:

a) “Typical AD syndrome” was classified by the AI tool when, in the tool report, amnestic cognitive impairment and disproportionate medial temporal lobe atrophy were reported;
b) Atypical AD syndrome, specifically Posterior Cortical Atrophy (PCA), was classified by the AI tool, when, in the tool report, visuospatial impairment and parieto-occipital atrophy were reported;
c) Atypical AD syndrome, specifically logopenic variant of Primary Progressive Aphasia (lvPPA) was classified by the AI tool, when, in the tool report, language impairment (ie, logopenic) and consistent focal atrophy in the dominant hemisphere were reported;
d) “Semantic PPA (svPPA)” was classified by the AI tool when, in the tool report, language impairment (ie, semantic) and consistent focal atrophy in the dominant hemisphere were reported;
e) “Agrammatic/nonfluent PPA (nfvPPA)” was classified by the AI tool when, in the tool report, language impairment (i.e., agrammatic or non-fluent) and consistent focal atrophy in the dominant hemisphere were reported.
f) “Behavioral variant of Frontotemporal dementia (bvFTD) or frontal variant AD (fvAD)” were classified by the AI tool when, in the tool report, frontal behavioral (i.e., disinhibition) or dysexecutive syndrome or both with frontotemporal atrophy were reported.
g) “No clear hypothesis” was classified when, in the tool report, cognitive impairment and MRI with negative or inconsistent results were reported. In these cases, the AI tool classified subjects based on the risk level (high risk, low risk) of having AD-dementia or converting to AD-dementia within 24-month, which is reported in the tool report.

The brain-volumetric features automatically processed by the AI tool were used to detect regional atrophy in specific brain regions when compared with normative percentiles (<10st percentile).

The clinical performance of the AI tool in clinical profiling was evaluated with respect to the biomarker-based diagnosis [CSF or PET according to the Intersocietal recommendation for each clinical syndrome (15)] in terms of classification accuracy for each stage (Performance I clinical profiling: AI tool vs. biomarkers in causal hypothesis).

#### 2.3.3 Subgroup analysis II: progression

The tool was used to classify subjects in the following distinct classes:

h) “Converter to AD-dementia” was classified by the AI tool when, in the tool report, an HR to convert to AD-dementia was reported;
i) “Non-Converter to AD-dementia” was classified by the AI tool when, in the tool report, a LR to convert to AD-dementia was reported;

The clinical performance of the AI tool in predicting, at baseline, the conversion of subjects to AD-dementia within 24-month was evaluated with respect to clinical diagnosis at 24-month follow-up, when 24-month follow-up was available, in terms of classification accuracy for each risk class (Performance II: Performance of AI tool vs. Clinical progression at 24-month follow-up).

#### 2.3.4 Statistical comparison with a similar tool

In order to compare TRACE4AD with a similar CE-marked tool, the 26 patients from IRCCS Policlinico San Donato-Università degli Studi di Milano, Milan, Italy (5 AD, 10 MCI, 11 HS) (Table 1, Center ID: PSD) were included in an independent analysis with the commercial tool Quantib ND (Quantib, Rotterdam, the Netherlands; now part of DeepHealth), available at that center. This tool allows the computation, from the brain 3D MRI (MP-RAGE) study of a subject, of volumetric measurements of lobes, cerebellum, and hippocampus (CSF and sum of gray and white matter) and provides a reference of these measurements with centile curves based on a population-derived sample of non-demented individuals (60). Similarly to TRACE4AD, as final output, for each subject, Quantib ND provides a report with the measured brain-volumetric features.

**Table 1:** Descriptive analysis of demographic variables and distribution of HS, SCI, MCI, and AD-dementia subjects at baseline and at 24-month follow-up across centers.

		Subjects *N* = 795										
						Baseline			24-month follow up			
Center ID	Country	Ethnic group (not Hispanic or Latino/Hispanic or Latino/NA)	Race group (Asian/Native Hawaiian or pacific islander/Black or African American/White/More than one race/NA)	*N*	Age (min–max)	HS	MCI	AD	HS	ncMCI	cMCI	AD
ADNI												
002	US/Canada	18/0/0	0/0/0/18/0/0	18	65–83	8	10	0	8	7	3	0
003	US/Canada	8/0/0	0/0/1/6/1/0	8	55–89	5	2	1	5	1	1	1
005	US/Canada	5/0/0	0/0/0/5/0/0	5	71–86	1	4	0	1	2	2	0
006	US/Canada	13/0/0	1/0/0/12/0/0	13	69–85	5	6	2	5	3	3	2
007	US/Canada	13/1/0	0/0/1/13/0/0	14	67–88	5	7	2	5	5	2	2
009	US/Canada	7/0/0	0/0/0/7/0/0	7	63–77	3	4	0	3	2	2	0
010	US/Canada	6/1/0	0/0/1/5/0/0	7	65–79	3	2	2	3	0	2	2
011	US/Canada	16/0/0	0/0/3/13/0/0	16	60–82	7	8	1	7	6	2	1
012	US/Canada	13/1/0	0/1/0/13/0/0	14	60–85	3	10	1	3	8	2	1
013	US/Canada	9/1/0	0/0/0/8/1/1	10	58–87	5	4	1	5	3	1	1
014	US/Canada	13/1/0	0/0/1/12/1/0	14	86–85	7	5	2	7	1	4	2
016	US/Canada	8/3/0	0/0/0/11/0/0	11	55–86	2	7	2	2	2	5	2
018	US/Canada	22/1/0	0/0/2/21/0/0	23	66–86	7	11	5	7	10	1	5
019	US/Canada	11/2/0	0/0/0/13/0/0	13	57–90	3	8	2	3	6	2	2
020	US/Canada	6/0/0	0/0/0/6/0/0	6	66–83	6	0	0	6	0	0	0
021	US/Canada	12/0/0	0/0/1/11/0/0	12	60–83	3	6	3	3	4	2	3
022	US/Canada	18/2/0	0/0/1/7/2/0	20	60–81	7	9	4	7	9	0	4
023	US/Canada	20/0/0	0/0/0/19/1/0	20	59–84	4	12	4	4	5	7	4
024	US/Canada	11/0/0	1/0/0/7/2/0	11	62–84	5	4	2	5	3	1	2
027	US/Canada	8/0/0	0/0/0/8/0/0	8	73–88	2	2	4	2	1	1	4
029	US/Canada	7/0/0	1/0/0/6/0/0	7	66–86	0	6	1	0	5	1	1
031	US/Canada	18/0/0	0/0/2/16/0/0	18	56–89	4	10	4	4	5	5	4
032	US/Canada	11/0/0	0/0/0/11/0/0	11	60–85	5	4	2	5	1	3	2
033	US/Canada	11/0/0	0/0/0/11/0/0	11	57–83	5	3	3	5	2	1	3
035	US/Canada	4/0/0	1/0/0/3/0/0	4	74–81	1	3	0	1	2	1	0
036	US/Canada	17/0/0	0/0/2/15/0/0	17	57–84	5	11	1	5	6	5	1
037	US/Canada	22/0/0	1/0/1/20/0/0	22	59–89	6	13	3	6	10	3	3
041	US/Canada	29/0/0	0/0/2/26/0/0	29	60–89	15	14	0	15	13	1	0
051	US/Canada	1/0/0	0/0/0/1/0/0	1	76–76	0	1	0	0	0	1	0
052	US/Canada	3/0/0	0/0/0/3/0/0	3	61–85	1	2	0	1	2	0	0
053	US/Canada	6/0/0	0/0/0/6/0/0	6	66–83	1	4	1	1	3	1	1
057	US/Canada	6/0/0	0/0/0/6/0/0	6	72–82	1	3	2	1	1	2	2
067	US/Canada	12/0/0	0/0/0/10/0/0	12	58–83	3	4	5	3	3	1	5
068	US/Canada	11/0/0	0/0/0/11/0/0	11	56–77	2	8	1	2	6	2	1
070	US/Canada	1/0/0	0/0/0/1/0/0	1	83–83	0	1	0	0	1	0	0
072	US/Canada	10/0/0	0/0/0/10/0/0	10	59–87	3	7	0	3	6	1	0
073	US/Canada	13/1/0	3/0/0/10/0/0	14	61–83	12	2	0	12	1	1	0
082	US/Canada	13/0/0	0/0/0/12/0/0	13	67–84	9	2	2	9	0	2	2
094	US/Canada	6/0/0	0/0/1/5/0/0	6	69–85	4	1	1	4	1	0	1
098	US/Canada	4/0/0	0/0/0/4/0/0	4	69–88	1	2	1	1	1	1	1
099	US/Canada	7/1/0	0/0/0/8/0/0	8	67–82	6	2	0	6	0	2	0
100	US/Canada	13/0/0	1/0/1/11/0/0	13	66–85	7	2	4	7	0	2	4
109	US/Canada	3/0/0	0/0/3/0/0/0	3	77–85	2	0	1	2	0	0	1
114	US/Canada	16/1/0	0/0/3/13/1/0	17	56–86	10	4	3	10	4	0	3
116	US/Canada	28/2/0	2/0/0/25/2/1	30	56–87	12	11	7	12	5	6	7
123	US/Canada	12/0/0	0/0/0/12/0/0	12	62–85	3	4	5	3	0	4	5
126	US/Canada	7/0/0	0/0/1/6/0/0	7	74–84	1	4	2	1	2	2	2
127	US/Canada	7/0/0	1/0/0/6/0/0	7	71–85	3	3	1	3	3	0	1
128	US/Canada	22/0/1	2/0/2/18/1/0	23	56–86	8	9	6	8	7	2	6
129	US/Canada	1/0/0	0/0/0/1/0/0	1	73–73	0	1	0	0	1	0	0
130	US/Canada	19/0/0	1/0/1/17/0/0	19	64–85	9	7	3	9	4	3	3
131	US/Canada	6/0/0	0/0/0/6/0/0	6	62–79	2	4	0	2	2	2	0
133	US/Canada	4/0/0	0/0/0/4/0/0	4	70–86	3	0	1	3	0	0	1
135	US/Canada	6/0/0	0/0/0/6/0/0	6	65–79	2	2	2	2	0	2	2
136	US/Canada	8/0/0	0/0/0/8/0/0	8	66–81	4	4	0	4	0	4	0
137	US/Canada	25/0/0	1/0/0/24/0/0	25	55–85	8	11	6	8	5	6	6
141	US/Canada	14/0/0	1/0/1/12/0/0	14	63–84	6	4	4	6	0	4	4
153	US/Canada	4/0/0	0/0/0/5/0/0	4	70–76	3	0	1	3	0	0	1
168	US/Canada	12/1/0	0/0/0/12/0/0	13	58–68	7	5	1	7	3	2	1
177	US/Canada	3/0/0	0/0/0/3/0/0	3	58–68	3	0	0	3	0	0	0
301	US/Canada	2/1/0	0/0/0/3/0/0	3	65–81	0	3	0	0	3	0	0
341	US/Canada	2/0/0	0/0/0/2/0/0	2	66–76	1	1	0	1	1	0	0
941	US/Canada	20/0/1	1/0/0/19/1/0	21	57–86	19	1	1	19	1	0	1
CDI	Italy (EU)	34/0/0	0/0/0/0/0/34	34	39–83	1	31	2	NA	NA	NA	NA
PSD	Italy (EU)	26/0/0	0/0/0/0/0/26	26	60–85	10	11	5	NA	NA	NA	NA
BP	Italy (EU)	30/0/0	0/0/0/0/0/30	30	67–88	1	15	14	NA	NA	NA	NA

ADNI, Alzheimer’s Disease Neuroimaging Initiative; HS, Healthy subjects; cMCI, converter-Mild Cognitive Impairment; nc-MCI, non converter-Mild Cognitive Impairment; AD, Alzheimer’s Disease; CDI, Centro Diagnostico Italiano; MCI, Mild Cognitive Impairment; NA, Not Available. The identification of ADNI centers is not made available from the ADNI protocol. CDI, Centro Diagnostico Italiano; PSD, IRCCS Policlinico San Donato; EU, Europe; BP, IRCCS Bonino-Pulejo.

One MRI per subject was processed by Quantib ND and compared with the TRACE4AD report to assess any diagnostic differences. This included: (1) assessing the correlation between the brain-volumetric features CSF, lobe, cerebellum, and hippocampus volumes measured by the two tools; (2) evaluating the agreement in the analysis of brain volumes affected by atrophy; and (3) comparing the diagnostic performance of the brain-volumetric features extracted by both tools in classifying HS, MCI, and AD. For these purposes, Spearman’s correlation coefficients were computed between the brain-volumetric features calculated by both the tools. Cohen’s *k* was computed to assess agreement in brain volumes atrophy analysis based on the two tools. ROC-AUC analysis with DeLong tests [‘pROC’, R package, IBM Inc. (61)] was used to compare the diagnostic performance in classifying HS, MCI, and AD based on the brain-volumetric features extracted by both the tools [as in (62)].

#### 2.3.5 Statistical distributions

The sociodemographic characteristics were presented using descriptive statistics. Continuous variables were reported as range (min to max) and categorical variables were presented as frequency and proportions (%).

The agreement in staging between AI tool and humans was computed using Cohen’s *k* in each staging class and in the overall subgroup.

The AI tool diagnostic performances were presented with mean value and 95% confidence intervals (CI), calculated using the Exact method.

Brain-volumetric features, cognitive measures, automatically processed by the AI tool, and CSF biomarkers were statistically reported according to the different stages, clinical syndromes and progression profiles and their subgroup analysis. Cognitive measures, brain-volumetric features, and CSF biomarkers were reported as mean ± standard deviation (SD). Normal distributions of quantitative variables were tested using the Shapiro–Wilk test. To assess differences between groups, a statistical analysis based on the null hypothesis significance test was applied. Normal distributed variables were tested using the parametric t-test, while for not normally distributed variables the non-parametric U Mann–Whitney test was used. The Bonferroni correction method was used to adjust for multiple comparisons.

Spearman’s correlation tests between brain-volumetric features, and CSF biomarkers (Aβ42, t-tau, and p-tau) were performed in its subgroup.

Spearman’s correlation coefficients were calculated between cognitive measures and brain-volumetric features were performed across the different stages.

The significance level adopted was 5% (*p* < 0.05), with 95% confidence intervals (CI). Data were analyzed using the RStudio (63) program version 2024.04.2.

## 3 Results

### 3.1 Study population

A total of 795 subjects were included: mean-age (calculated on 761 subjects) 73.54 ± 7.52; sex (%) 52/45/3, males/females/missing; mean education (y) (calculated on 705 subjects): 16.38 ± 2.70; ethnicity (%): 2.6/97.1/0.4, Hispanic-or-Latino/not-Hispanic-or-Latino/missing; racial category (%): 2.6/0.1/4.1/81.4/1.8/10, Asian/native Hawaiian or pacific islander/black or African American/white/more than one race/missing; primary language (%): 88.7/0.5/1/9.8, English/Spanish/others/missing; handness (%): 84/6/10 right/left/missing). The distribution of HS, SCI, MCI, and AD-dementia subjects at baseline and at 24-month follow-up is shown in Table 1 across the 66 centers.

### 3.2 Clinical data collection

#### 3.2.1 MRI studies

Among the 795 participants (Whole cohort), all subjects performed 3D T1-weighted MP-RAGE MRI at baseline: 391 at 1.5 T, and 390 at 3 T. 705 subjects had 3D T1-weighted MP-RAGE MRI at both baseline and 24-month follow-up (Subgroup II).

#### 3.2.2 Neuropsychological studies

Among the 795 participants, 426 subjects had completed all the neuropsychological test scores (in addition to 3D T1-weighted MP-RAGE MRI) at baseline (Subgroup 0), and 341 at both baseline and clinical follow-up (Subgroup IV).

#### 3.2.3 First line biomarkers

Among the 795 participants, 485 subjects underwent lumbar puncture at baseline: 482 subjects had all three proteins measured (Subgroup III) (two subjects had only Aβ-42 concentrations, one subject had only Aβ-42 and t-tau proteins concentration).

159 subjects had neuroimaging studies, neuropsychological tests and biological biomarkers (CSF or PET) (Subgroup Ia).

### 3.3 Data processing

All subjects’ MRI data (795 subjects) and neuropsychological data (341 subjects) were safely processed by TRACE4AD.

#### 3.3.1 Subgroup analysis 0: staging

In order to evaluate the AI-tool performance with respect to subjects’ staging (Performance 0), 426 subjects were considered and re-staged by the software, being already clinically staged by clinicians at baseline at their sites (Subgroup 0: *N =* 426). In Table 2, the agreement is presented for different stages (HS/SCI/WW, MCI/MD, MSD).

**Table 2:** TRACE4AD staging performance compared to clinical staging.

Performance 0 Staging: Agreement of AI tool vs. clinicians (Subgroup 0: *N* = 426)			
	MSD	MCI/MD	HS/SCI/WW
	*N* = 95	*N* = 219	*N* = 112
Cohen’s *k*	0.90	0.70	0.81
3-classes Cohen’s *k* 0.64			

Inter-rater agreement (Cohen’s *k*) between AI and clinicians was substantial for both MCI/MD-vs-rest (0.70) and HS/SCI/WW-vs-rest (0.81) classification, almost perfect for MSD-vs-rest (0.90) classification. Also, the inter-rater agreement between AI and clinicians for 3-classes categorization (MSD vs. MCI/MD vs. HS/SCI/WW) was substantial (Cohen’s *k* = 0.64). However, the AI tool restaged HS as MCI in 42% cases (47/112). Among these subjects, based on the detailed neuropsychological assessment including eight neuropsychological tests (see Section 2.2.1), 42/47 had a memory impairment, 1/47 had functional impairment, and 4/47 had a significant cognitive impairment. Additionally, looking at biological findings,18 had biomarkers available for a biological diagnosis: 7 on 18 (39%, about ⅓) were AD, 3 had Aβ42 + t-tau- (1 with negative [^18^F]FDG PET) (HS/SCI/WW), 1 had Aβ42- t-tau+ with negative [^18^F]FDG PET (HS/SCI/WW), 7 had excluded AD.

#### 3.3.2 Subgroup analysis I: clinical profiling, clinical diagnosis and causal hypothesis

In order to evaluate the AI-tool performance with respect to clinical profile for clinical syndrome classification, clinical diagnosis and causal hypothesis at baseline (Performance I), 130 subjects from Subgroup Ia staged by AI as MCI-MD (64) or MSD (48) with available biomarkers were considered (Subgroup Ib: *N* = 130). In Table 3, the clinical syndrome classification, clinical diagnosis and causal hypothesis at baseline using AI tool is presented.

**Table 3:** Clinical syndrome classification, clinical diagnosis and causal hypothesis at baseline using AI tool.

AI-based assessment					
Clinical syndrome	Amnestic cognitive impairment and disproportionate medial temporal lobe atrophy	Visuospatial impairment and parietooccipital atrophy	Language impairment (i.e., logopenic, agrammatic or non-fluent, or semantic) and consistent focal atrophy in the dominant hemisphere	Frontal behavioral or dysexecutive syndrome or both with frontotemporal atrophy	No clear hypothesis
Clinical diagnosis	Typical AD syndrome *N =* 79	PCA *N =* 2	lv-PPA *N =* 15	bvFTD or fvAD *N =* 9	No clear hypothesis *N =* 25
Causal hypothesis	Suspected AD *N =* 96			Suspected FTLD *N =* 9	No clear hypothesis *N =* 25
*Risk of progression to AD-dementia*				HR *N =* 8 LR *N =* 1	HR *N =* 7 LR *N =* 18
Causal Hypothesis	Suspected AD *N =* 96 + 8 + 7			Suspected FTLD *N =* 1	No clear hypothesis *N =* 18
CSF Biomarkers + [^18^F]FDG PET	t-tau/Aβ42 + *N =* 87 + 3 + 3 + 5 t-tau/Aβ42- *N =* 7 + 3 Aβ42- t-tau+, [^18^F]FDG PET+ *N =* 1 [^18^F]FDG PET+ *N =* 2			t-tau/Aβ42- *N =* 1	t-tau/Aβ42 + *N =* 8 t-tau/Aβ42- *N =* 1 + 7 Aβ42 + t-tau-, [^18^F]FDG PET- *N =* 1 Aβ42- t-tau+, [^18^F]FDG PET- = 1
TOT = 130	*N =* 111			*N =* 1	*N =* 18
Accuracy	101/111 = 91.0%			1/1 = 100%	

Regarding the results, AI classified 79 subjects based on MRI and neuropsychological markers with clinical syndrome compatible with typical AD (amnestic cognitive impairment and disproportionate medial temporal lobe atrophy), two subjects with clinical syndrome compatible with PCA (visuospatial impairment and parieto-occipital atrophy), 15 subjects with clinical syndrome compatible lv-PPA (language impairment and consistent focal atrophy in the dominant hemisphere), nine subjects with clinical syndrome compatible bvFTD or fvAD according to intersocietal classification (frontal behavioral or dysexecutive syndrome or both with fronto-temporal atrophy); 25 subjects were classified as no clear hypothesis. Related causal hypotheses identified by AI were suspected AD for 96 subjects, frontotemporal lobe degeneration (FTLD) for nine subjects, and no clear hypothesis for 25 subjects. Considering the AI-based risk of progression to AD within 24 months, eight subjects previously classified as “bvFTD or fvAD” were re-classified as “suspected AD”; seven subjects previously classified as “no clear hypothesis” were re-classified as “suspected AD.”

Considering CSF biomarkers and [^18^F]FDG PET (reference standard for the biological diagnosis):

a) among the 96 + 7 subjects classified by AI as suspected AD, 93 had a biological diagnosis of AD (t-tau/Aβ42+), 3 had positive [^18^F]FDG PET for AD, while 7 had CSF biomarkers that excluded AD (t-tau/Aβ42-);
b) among the nine subjects classified by AI as suspected FTLD, 4 had CSF biomarkers that excluded AD (t-tau/Aβ42-), while 5 had a biological diagnosis of AD (t-tau/Aβ42+);
c) among the 18 subjects classified by AI as “No clear hypothesis,” 8 had a biological diagnosis of AD (t-tau/Aβ42+), 8 had CSF biomarkers that excluded AD (t-tau/Aβ42-), 1 had Aβ42 + t-tau- but negative [^18^F]FDG PET for AD, and 1 had Aβ42- t-tau+ but negative [^18^F]FDG PET for AD.

Considering all subjects classified by AI as suspected AD or suspected FTLD, AI accuracy in comparison with biomarker-based diagnosis (CSF or PET) was 89.3% (100/112).

Overall, in Table 4, AI tool clinical performances in AD vs. FTLD clinical syndrome (excluded subjects with no clear hypothesis) are presented in terms of positive predictive value (PPV), negative predictive value (NPV), and accuracy in classifying AD vs. FTLD (CSF or PET biomarkers as reference standards).

**Table 4:** AI tool clinical performances in AD vs. FTLD clinical syndrome (excluded subjects with no clear hypothesis) in classifying AD vs. FTLD using CSF or PET biomarkers as reference standards.

Performance I: Performance of AI tool vs. biomarkers in causal hypothesis (Subgroup I: *N =* 130–18* = 112)		
AD FTLD		
PPV	NPV	Accuracy
91% [84–96%95 CI]	100%	91% [84–96%95 CI]
*N =* 101/111	*N =* 1/1	*N =* 102/112

ROC-AUC, Receiver Operating Characteristics-Area Under Curve; PPV, Positive Predictive Value; NPV, Negative Predictive Value; %95CI, Confidence Interval 95%; *no clear hypothesis.

Sensitivity and specificity were not calculated because subjects classified by AI as suspected AD, but with CSF biomarkers that excluded AD, do not necessarily belong to the “suspected FTLD” causal hypothesis. Calculating these metrics under such conditions could lead to misleading conclusions.

#### 3.3.3 Subgroup analysis II: progression

In order to evaluate the AI tool performance to predict clinical progression of dementia at 24-month follow-up (Performance II), 705 subjects with 24-month follow-up were considered (Subgroup II: *N =* 705). In Tables 5, 6, AI-tool clinical performances in clinical progression (sensitivity, specificity, accuracy, ROC-AUC) are presented.

**Table 5:** Performance of the AI tool in clinical progression at 24-month follow-up using MRI data, compared to clinicians.

Performance II: Performance of AI tool vs. Clinical progression at 24-month follow-up (Subgroup II: *N =* 705)			
	Converter to AD-dementia *N =* 272	Non converter to AD-dementia *N =* 433	
Sensitivity	Specificity	Accuracy	ROC-AUC
79% [73–83%95 CI]	81% [77–84%95 CI]	80% [77–83%95 CI]	85% [82–88%95 CI]
N. 180/272	N. 384/433	N. 384/705	

ROC-AUC, Receiver Operating Characteristics-Area Under Curve; PPV, Positive Predictive Value; NPV, Negative Predictive Value; %95CI, Confidence Interval 95%.

**Table 6:** Performance of the AI tool in clinical progression at 24-month follow-up using MRI data and cognitive measures, compared to clinicians.

Performance II: Accuracy of AI tool vs. Clinical progression at 24-month follow-up (Subgroup IV: *N =* 341)			
	Converter to AD-dementia *N =* 167	Non converter to AD-dementia *N =* 174	
Sensitivity	Specificity	Accuracy	ROC-AUC
89% [82–93%95 CI]	82% [76–87%95 CI]	85% [80–88%95 CI]	83% [79–87%95 CI]
N. 132/149	157/192	N. 289/341	

ROC-AUC, Receiver Operating Characteristics-Area Under Curve; PPV, Positive Predictive Value; NPV, Negative Predictive Value; %95CI, Confidence Interval 95%.

Two hundred seventy-two subjects were predicted as LR, and 433 as HR to convert to AD-dementia at 24-month follow-up by TRACE4AD using MRI data: sensitivity, specificity, accuracy, and ROC-AUC of the tool in predicting subjects converting or not to AD-dementia within 24-month compared to clinical diagnosis were 79% [74–84%95 CI], 81% [77–85%95 CI], 80% [77–83%95 CI], and 85% [82–87%95 CI], respectively. To be noted, the AI tool has a high ROC-AUC (85%), sensitivity, and specificity (80%), thus it is useful to predict conversion or not to AD-dementia and to support clinical profiling at 24-month follow-up.

One hundred seventy-four subjects were predicted as LR, and 167 as HR to convert to AD-dementia at 24-month follow-up by TRACE4AD using MRI and neuropsychological data: sensitivity, specificity, accuracy, and ROC-AUC of the tool in predicting subjects converting or not to AD-dementia within 24-month compared to clinical diagnosis were 89% [84–94%95 CI], 82% [77–87%95 CI], 85% [81–89%95 CI], and 83% [79–87%95 CI], respectively.

#### 3.3.4 Statistical comparison between different tools

In Table 7, Quantib ND and TRACE4AD correlation results are presented; normative regional data interpreted for diagnosis showed strong to very strong and statistically significant correlation (rs = 0.70–0.94).

**Table 7:** Spearman’s correlation between brain volumetric features extracted with either Quantib or TRACE4AD.

	Spearman’s rho	*p*-value
Whole brain volume	0.94	*p* < 0.001***
TIV	0.93	*p* < 0.001***
CSF	0.80	*p* < 0.001***
Frontal lobe	0.88	*p* < 0.001***
Occipital lobe	0.78	*p* < 0.001***
Temporal lobe	0.92	*p* < 0.001***
Parietal lobe	0.92	*p* < 0.001***
Cerebellum	0.70	*p* < 0.001***
Hippocampus	0.88	*p* < 0.001***

CSF, cerebrospinal fluid; TIV, total intracranial volume. * for 0.01 < *p*-value < 0.05, ** for *p*-value < 0.01, *** for *p*-value < 0.001.

Table 8 shows the Quantib ND and TRACE4AD atrophy analysis agreement. The two tools demonstrated a fair and statistically significant agreement for the occipital lobe (whole: *k* = 0.35, *p* = 0.02; left: *k* = 0.40, *p* = 0.01). Similarly, a moderate-to-substantial and statistically significant agreement was observed for the temporal lobe (whole: *k* = 0.61, *p* = 0.001; left: *k* = 0.40, *p* < 0.01; right: *k* = 0.57, *p* < 0.01) and for the hippocampus (whole: *k* = 0.47, *p* = 0.01; left: *k* = 0.43, *p* = 0.02; right: *k* = 0.30, *p* = 0.03).

**Table 8:** Atrophy analysis agreement between TRACE4AD and Quantib ND.

	Cohen’s *k*	*p*-value
Frontal lobe		
Whole	0.15	0.35
Left	0.28	0.10
Right	0.08	0.65
Parietal lobe		
Whole	−0.07	0.70
Left	−0.03	0.88
Right	0.03	0.88
Occipital lobe		
Whole	0.35	0.02*
Left	0.40	0.01*
Right	0.20	0.09
Temporal lobe		
Whole	0.61	0.001***
Left	0.43	*p* < 0.01**
Right	0.57	*p* < 0.01**
Hippocampus		
Whole	0.47	0.01**
Left	0.43	0.02*
Right	0.30	0.03*

* for 0.01 < *p*-value < 0.05, ** for *p*-value < 0.01, *** for *p*-value < 0.001.

In Table 9, the diagnostic performance comparisons are presented. Quantib ND and TRACE4AD brain-volumetric features were not statistically different in differentiating HS, MCI, and AD (*p* > 0.05).

**Table 9:** Diagnostic performance of each brain-volumetric feature according to AI extraction tool.

	TRACE4AD	Quantib ND	
Brain-volumetric features	ROC-AUC [%95CI]	ROC-AUC [%95CI]	*p*-value
Whole brain [mL]	0.77 [0.56–0.99]	0.70 [0.46–0.94]	0.15
TIV [mL]	0.65 [0.40–0.89]	0.66 [0.41–0.92]	0.80
CSF [mL]	0.68 [0.44–0.92]	0.65 [0.40–0.89]	0.68
Frontal lobe [mL]	0.75 [0.52–0.98]	0.67 [0.42–0.93]	0.22
Parietal lobe [mL]	0.75 [0.54–0.97]	0.67 [0.43–0.92]	0.15
Occipital lobe [mL]	0.73 [0.48–0.95]	0.72 [0.49–0.95]	0.94
Temporal lobe [mL]	0.76 [0.54–0.99]	0.69 [0.45–0.92]	0.21
Cerebellum [mL]	0.79 [0.59–0.99]	0.78 [0.58–0.98]	0.92
Hippocampus [mL]	0.79 [0.57–1.00]	0.69 [0.44–0.93]	0.09

CSF, cerebrospinal fluid; TIV, total intracranial volume.

#### 3.3.5 Statistical distributions

The brain-volumetric features and the cognitive features automatically processed by the AI tool are reported in Tables 10–14 according, respectively, to different stages of AD clinical syndromes, progression to AD-dementia and their subgroup analysis.

**Table 10:** Descriptive analysis of cognitive measures, for subjects with AD clinical syndromes at different stages (Subgroup I: *N =* 130).

	MCI/MD (*N =* 79)	MSD (*N =* 51)			
Cognitive measures	Mean ± SD	Mean ± SD	Difference (%)	*p*-value	*p*-value☨
MMSE ‡	27.48 ± 1.77	24.14 ± 1.97	12.15	*p* < 0.001***	*p* < 0.001***
AVLT					
*Trial1 ‡*	4.14 ± 1.45	3.51 ± 1.45	15.22	0.02*	1
*Trial1 errors ‡*	0.48 ± 0.78	0.37 ± 0.77	22.92	0.28	1
*Trial2 ‡*	5.52 ± 1.77	4.49 ± 1.74	18.66	*p* < 0.01**	0.08
*Trial2 errors ‡*	0.57 ± 0.98	0.53 ± 1.10	7.02	0.67	1
*Trial3 ‡*	6.61 ± 2.01	5.28 ± 1.89	20.12	*p* < 0.001***	0.02*
*Trial3 errors ‡*	0.63 ± 0.89	0.61 ± 0.90	3.17	0.92	1
*Trial4 ‡*	6.92 ± 2.16	5.61 ± 1.96	18.93	*p* < 0.01**	0.06
*Trial4 errors ‡*	0.48 ± 0.78	0.53 ± 0.86	−10.42	0.72	1
*Trial5 ‡*	7.43 ± 2.44	5.47 ± 2.22	26.38	*p* < 0.001***	*p* < 0.01**
*Trial5 errors ‡*	0.49 ± 0.81	0.39 ± 0.72	20.41	0.49	1
*Trial6 ‡*	3.56 ± 2.59	2.16 ± 2.04	39.33	*p* < 0.01**	0.07
*Trial6 errors ‡*	1.00 ± 1.26	1.29 ± 1.50	−29.00	0.31	1
*Delayed ‡*	2.33 ± 2.31	1.53 ± 2.60	34.33	0.01*	0.59
*Delayed errors ‡*	1.77 ± 2.00	1.18 ± 1.97	33.33	*p* < 0.01**	0.44
*Recognitions ‡*	9.68 ± 3.52	8.08 ± 3.98	16.53	0.02*	1
*Recognitions errors ‡*	1.91 ± 2.05	2.63 ± 2.08	−37.70	0.03*	1
Digit Span Forward ‡	6.68 ± 1.06	6.45 ± 1.08	3.44	0.29	1
Digit Span Backward ‡	4.80 ± 1.16	4.12 ± 1.11	14.17	*p* < 0.01**	0.09
TMT-A					
*Time taken ‡*	43.00 ± 21.10	64.04 ± 36.76	−48.93	*p* < 0.001***	0.01*
*Committed errors ‡*	0.06 ± 0.29	0.23 ± 0.62	−283.33	0.04*	1.00
*Omission errors ‡*	0.01 ± 0.11	0.08 ± 0.44	−700.00	0.33	1.00
TMT-B					
*Time taken ‡*	115.18 ± 59.08	211.20 ± 86.69	−83.37	*p* < 0.001***	*p* < 0.001***
*Committed errors ‡*	0.77 ± 1.26	1.96 ± 2.02	−154.55	*p* < 0.001***	*p* < 0.01**
*Omission errors ‡*	0.14 ± 0.62	4.51 ± 7.09	−3,121.43	*p* < 0.001***	*p* < 0.001***
Clock					
*Contour ‡*	0.98 ± 0.16	0.98 ± 0.14	0.00	0.84	1.00
*Number order ‡*	0.73 ± 0.44	0.47 ± 0.50	35.62	*p* < 0.01**	0.14
*Numbers present ‡*	0.84 ± 0.37	0.71 ± 0.46	15.48	0.08	1.00
*Hands ‡*	0.95 ± 0.22	0.84 ± 0.37	11.58	0.04*	1.00
*Time signed ‡*	0.66 ± 0.48	0.45 ± 0.50	31.82	0.02*	1.00
*Total score ‡*	4.15 ± 1.03	3.45 ± 1.30	16.87	*p* < 0.01**	0.06
Symbol digit	40.19 ± 9.91	28.67 ± 11.43	28.66	*p* < 0.001***	*p* < 0.001***
Category fluency					
*Animals*	16.08 ± 4.70	13.94 ± 4.88	13.31	0.02*	0.83
*Animals perseveration ‡*	1.23 ± 1.63	1.49 ± 1.64	−21.14	0.23	1.00
*Animals intrusion ‡*	0.06 ± 0.29	0.20 ± 0.85	−233.33	0.79	1.00
*Vegetables*	11.89 ± 3.42	8.45 ± 3.67	28.93	*p* < 0.001***	*p* < 0.001***
*Vegetables perseveration ‡*	0.57 ± 0.80	0.51 ± 1.08	10.53	0.21	1.00
*Vegetables intrusion ‡*	0.51 ± 1.15	0.80 ± 1.72	−56.86	0.41	1.00
Boston Naming Test					
*Correct spontaneous answers ‡*	25.86 ± 3.52	23.41 ± 5.49	9.47	*p* < 0.01**	0.33
*Semantic cues ‡*	2.09 ± 3.16	3.02 ± 3.75	−44.50	0.11	1.00
*Correct answer after semantic cue ‡*	0.27 ± 0.69	0.61 ± 1.46	−125.93	0.05	1.00
*Phonological cues ‡*	3.73 ± 3.40	5.72 ± 5.58	−53.35	0.04*	1.00
*Correct answer after phonological cue ‡*	0.27 ± 0.69	0.61 ± 1.46	−125.93	0.05	1.00
*Total score ‡*	26.13 ± 3.43	24.02 ± 5.47	8.08	0.02*	1.00
FAQ					
*Finances ‡*	0.90 ± 1.46	3.00 ± 1.81	−233.33	*p* < 0.001***	*p* < 0.001***
*Bills ‡*	0.85 ± 1.41	3.37 ± 1.61	−296.47	*p* < 0.001***	*p* < 0.001***
*Buying ‡*	0.62 ± 1.27	2.51 ± 1.81	−304.84	*p* < 0.001***	*p* < 0.001***
*Social life ‡*	0.51 ± 1.12	1.67 ± 1.67	−227.45	*p* < 0.001***	*p* < 0.001***
*Housekeeping ‡*	0.22 ± 0.76	1.00 ± 1.62	−354.55	*p* < 0.001***	0.04*
*Cooking ‡*	0.52 ± 0.97	1.80 ± 1.70	−246.15	*p* < 0.001***	*p* < 0.001***
*Keeping up with external events ‡*	0.48 ± 1.19	2.12 ± 1.81	−341.67	*p* < 0.001***	*p* < 0.001***
*Entertainment and learning ‡*	0.56 ± 1.22	1.51 ± 1.65	−169.64	*p* < 0.001***	0.02*
*Memory ‡*	1.37 ± 1.61	3.69 ± 1.05	−169.34	*p* < 0.001***	*p* < 0.001***
*Transports ‡*	0.48 ± 1.15	2.51 ± 2.19	−422.92	*p* < 0.001***	*p* < 0.001***
*Total score ‡*	2.48 ± 3.66	11.04 ± 6.40	−345.16	*p* < 0.001***	*p* < 0.001***

☨Bonferroni correction.

‡Non-parametric test.

SD, Standard deviation; MCI, Mild Cognitive Impairment; MD, Mild Dementia; MSD, Moderate-to-severe dementia; MMSE, Mini-Mental State Examination; AVLT, Auditory Verbal Learning Test; TMT, Trail Making Test; FAQ, Functional Assessment Questionnaire.

* for 0.01 < *p*-value < 0.05, ** for *p*-value < 0.01, *** for *p*-value < 0.001.

**Table 11:** Descriptive analysis of brain-volumetric features for subjects with AD clinical syndromes at different stages (Subgroup I: *N =* 130).

	MCI/MD (*N =* 79)	MSD (*N =* 51)			
Brain-volumetric features	Mean ± SD	Mean ± SD	Difference (%)	*p*-value	*p*-value☨
whole brain total volume	1,393.58 ± 86.72	1,338.75 ± 89.36	3.93	*p* < 0.001***	0.07
whole brain perc over tiv	70.47 ± 4.32	67.74 ± 4.43	3.87	*p* < 0.001***	0.06
whole brain rx	700.72 ± 42.94	671.11 ± 44.99	4.23	*p* < 0.001***	0.03*
whole brain lx	692.85 ± 44.53	667.64 ± 45.68	3.64	*p* < 0.01**	0.20
whole brain asymmetry index	0.57 ± 0.85	0.26 ± 1.15	54.39	0.10	1.00
gray matter total volume	731.80 ± 46.72	697.54 ± 51.09	4.68	*p* < 0.001***	0.02*
gray matter perc over tiv	37.01 ± 2.34	35.30 ± 2.53	4.62	*p* < 0.001***	0.02*
gray matter rx	368.07 ± 23.34	349.20 ± 26.75	5.13	*p* < 0.001***	*p* < 0.01**
gray matter lx	363.74 ± 24.06	348.34 ± 25.56	4.23	*p* < 0.001***	0.07
gray matter asymmetry index ‡	−0.60 ± 1.12	−0.11 ± 1.65	81.67	0.06	1.00
white matter total volume	661.78 ± 54.48	641.21 ± 54.30	3.11	0.04*	1.00
white matter perc over tiv	33.47 ± 2.73	32.45 ± 2.72	3.05	0.04*	1.00
white matter rx	332.66 ± 26.90	321.92 ± 26.70	3.23	0.03*	1.00
white matter lx	329.12 ± 27.87	319.30 ± 27.82	2.98	0.05	1.00
white matter asymmetry index	−0.55 ± 0.85	−0.43 ± 0.77	21.82	0.38	1.00
csf total volume	583.80 ± 84.91	637.32 ± 86.48	−9.17	*p* < 0.001***	0.06
csf perc over tiv	29.53 ± 4.32	32.26 ± 4.43	−9.24	*p* < 0.001***	0.06
tiv total volume ‡	1,977.37 ± 7.34	1,976.08 ± 7.89	0.07	0.35	1.00
cerebellum total volume	102.68 ± 11.22	101.25 ± 11.00	1.39	0.47	1.00
cerebellum rx	50.95 ± 5.64	50.29 ± 5.49	1.30	0.51	1.00
cerebellum lx	51.73 ± 5.69	50.96 ± 5.60	1.49	0.45	1.00
cerebellum asymmetry index	−0.76 ± 1.58	−0.66 ± 1.40	13.16	0.70	1.00
insula total volume	18.21 ± 1.82	17.58 ± 2.08	3.46	0.08	1.00
insula rx	8.96 ± 0.91	8.59 ± 1.07	4.13	0.04*	1.00
insula lx	9.25 ± 0.95	8.99 ± 1.06	2.81	0.15	1.00
insula asymmetry index	−1.60 ± 2.08	−2.34 ± 2.62	−46.25	0.09	1.00
cingulate cortex total volume	30.45 ± 2.49	29.22 ± 2.95	4.04	0.01*	1.00
cingulate cortex rx	15.34 ± 1.32	14.80 ± 1.69	3.52	0.06	1.00
cingulate cortex lx	15.11 ± 1.34	14.41 ± 1.41	4.63	*p* < 0.01**	0.50
cingulate cortex asymmetry index ‡	0.78 ± 3.16	1.23 ± 3.56	−57.69	0.18	1.00
hippocampus total volume	8.13 ± 1.43	7.36 ± 1.53	9.47	*p* < 0.01**	0.38
hippocampus rx	3.97 ± 0.76	3.55 ± 0.78	10.58	*p* < 0.01**	0.20
hippocampus lx	4.16 ± 0.72	3.81 ± 0.81	8.41	0.01*	1.00
hippocampus asymmetry index	−2.47 ± 4.76	−3.72 ± 5.95	−50.61	0.21	1.00
parahippocampus total volume	8.08 ± 1.04	7.54 ± 0.94	6.68	*p* < 0.01**	0.23
parahippocampus rx	4.39 ± 0.59	4.05 ± 0.55	7.74	*p* < 0.01**	0.10
parahippocampus lx	3.69 ± 0.53	3.50 ± 0.44	5.15	0.02*	1.00
parahippocampus asymmetry index	8.56 ± 5.16	7.24 ± 4.67	15.42	0.13	1.00
amygdala total volume	2.51 ± 0.39	2.34 ± 0.36	6.77	0.01*	0.83
amygdala rx	1.24 ± 0.20	1.16 ± 0.18	6.45	0.02*	1.00
amygdala lx	1.27 ± 0.21	1.18 ± 0.22	7.09	0.02*	1.00
amygdala asymmetry index	−1.29 ± 5.54	−0.67 ± 7.22	48.06	0.60	1.00
ventral striatum total volume	1.79 ± 0.23	1.78 ± 0.22	0.56	0.68	1.00
ventral striatum rx	0.85 ± 0.12	0.84 ± 0.11	1.18	0.69	1.00
ventral striatum lx	0.94 ± 0.12	0.93 ± 0.12	1.06	0.70	1.00
ventral striatum asymmetry index ‡	−5.07 ± 3.69	−5.11 ± 3.44	−0.79	0.99	1.00
thalamus total volume	10.35 ± 1.52	9.84 ± 1.81	4.93	0.10	1.00
thalamus rx	5.38 ± 0.80	5.12 ± 0.98	4.83	0.11	1.00
thalamus lx	4.96 ± 0.74	4.73 ± 0.87	4.64	0.12	1.00
thalamus asymmetry index ‡	4.11 ± 3.12	3.87 ± 4.34	5.84	0.81	1.00
precuneus total volume	23.12 ± 2.32	21.82 ± 2.41	5.62	*p* < 0.01**	0.25
precuneus rx	11.45 ± 1.24	10.78 ± 1.34	5.85	*p* < 0.01**	0.39
precuneus lx	11.67 ± 1.23	11.05 ± 1.20	5.31	*p* < 0.01**	0.42
precuneus asymmetry index	−0.95 ± 3.59	−1.35 ± 3.85	−42.11	0.55	1.00
ft cortex total volume	288.49 ± 20.38	272.06 ± 23.09	5.70	*p* < 0.001***	*p* < 0.01**
ft cortex rx ‡	145.44 ± 10.23	136.48 ± 12.11	6.16	*p* < 0.001***	0.01*
ft cortex lx	143.05 ± 10.49	135.58 ± 11.63	5.22	*p* < 0.001***	0.03*
ft cortex asymmetry index ‡	0.84 ± 1.31	0.31 ± 2.08	63.10	0.12	1.00
mt cortex total volume	16.21 ± 2.38	14.90 ± 2.40	8.08	*p* < 0.01**	0.23
mt cortex rx	8.36 ± 1.29	7.59 ± 1.29	9.21	*p* < 0.01**	0.11
mt cortex lx	7.85 ± 1.19	7.31 ± 1.20	6.88	0.01*	1.00
mt cortex asymmetry index	3.05 ± 4.37	1.83 ± 4.86	40.00	0.15	1.00
po cortex total volume	123.91 ± 9.99	115.61 ± 12.04	6.70	*p* < 0.001***	*p* < 0.01**
po cortex rx	58.59 ± 4.63	54.27 ± 6.03	7.37	*p* < 0.001***	*p* < 0.01**
po cortex lx	65.32 ± 5.61	61.33 ± 6.52	6.11	*p* < 0.001***	0.04*
po cortex asymmetry index	−5.40 ± 1.90	−6.13 ± 3.11	−13.52	0.14	1.00

☨Bonferroni correction.

‡Non-parametric test.

SD, Standard deviation; Difference (%), Percentage difference; MCI, Mild Cognitive Impairment; MD, Mild Dementia; MSD, Moderate-to-severe dementia; rx, right; lx, left; CSF, cerebrospinal fluid; tiv, Total Intracranial Volume; ft, fronto-temporal: frontal lobe + temporal lobe + anterior cingulate cortex; mt, mediotemporal; po, parieto-occipital: parietal lobe + occipital lobe.

* for 0.01 < *p*-value < 0.05, ** for *p*-value < 0.01, *** for *p*-value < 0.001.

**Table 12:** Descriptive analysis of CSF biomarkers, for subjects with AD clinical syndromes at different stages (Subgroup I: *N =* 130).

	MCI/MD (*N =* 79)	MSD (*N =* 51)			
CSF biomarkers	Mean ± SD	Mean ± SD	Difference (%)	*p*-value	*p*-value☨
Aβ42‡	796.53 ± 403.38	686.69 ± 428.29	13.79	0.03*	0.13
t-tau ‡	322.93 ± 123.38	361.32 ± 134.24	−11.89	0.06	0.24
p-tau ‡	31.97 ± 14.14	36.54 ± 15.88	−14.29	0.07	0.28
t-tau/Aβ42 ‡	0.51 ± 0.28	0.63 ± 0.29	−23.53	0.02*	0.06

☨Bonferroni correction.

‡Non-parametric test.

SD, Standard deviation; MCI, Mild Cognitive Impairment; MD, Mild Dementia; MSD, Moderate-to-severe dementia; CSF, cerebrospinal fluid; Aβ42, amyloid-beta 42; t-tau, total-tau; p-tau, phosphorylated-tau.

* for 0.01 < *p*-value < 0.05, ** for *p*-value < 0.01, *** for *p*-value < 0.001.

**Table 13:** Descriptive analysis of cognitive and brain-volumetric features according to the AI-tool predicted risk of conversion or not to AD-dementia within 24-month using MRI and cognitive data (Subgroup IV: *N =* 341).

	Low risk (*N =* 174)	High risk (*N =* 167)		
	Mean ± SD	Mean ± SD	*p*-value	*p*-value☨
Brain-volumetric features				
whole brain total volume	1,413.51 ± 84.52	1,339.25 ± 88.43	*p* < 0.001***	*p* < 0.001***
whole brain perc over tiv	71.47 ± 4.22	67.84 ± 4.38	*p* < 0.001***	*p* < 0.001***
whole brain rx	710.28 ± 42.58	672.95 ± 45.12	*p* < 0.001***	*p* < 0.001***
whole brain lx	703.24 ± 42.39	666.30 ± 44.74	*p* < 0.001***	*p* < 0.001***
whole brain asymmetry index ‡	0.50 ± 0.63	0.50 ± 1.19	0.75	1.00
gray matter total volume	743.43 ± 48.84	700.34 ± 49.78	*p* < 0.001***	*p* < 0.001***
gray matter perc over tiv	37.59 ± 2.45	35.48 ± 2.48	*p* < 0.001***	*p* < 0.001***
gray matter rx	373.26 ± 24.64	351.77 ± 25.65	*p* < 0.001***	*p* < 0.001***
gray matter lx	370.17 ± 24.63	348.57 ± 25.51	*p* < 0.001***	*p* < 0.001***
gray matter asymmetry index ‡	−0.42 ± 0.88	−0.46 ± 1.72	0.92	1.00
white matter total volume	670.08 ± 52.35	638.91 ± 54.16	*p* < 0.001***	*p* < 0.001***
white matter perc over tiv	33.88 ± 2.63	32.36 ± 2.70	*p* < 0.001***	*p* < 0.001***
white matter rx	337.01 ± 26.17	321.18 ± 27.12	*p* < 0.001***	*p* < 0.001***
white matter lx	333.06 ± 26.40	317.73 ± 27.36	*p* < 0.001***	*p* < 0.001***
white matter asymmetry index	−0.60 ± 0.70	−0.55 ± 0.92	0.60	1.00
csf total volume	564.18 ± 83.24	634.68 ± 85.78	*p* < 0.001***	*p* < 0.001***
csf perc over tiv	28.53 ± 4.22	32.16 ± 4.38	*p* < 0.001***	*p* < 0.001***
tiv total volume ‡	1,977.69 ± 7.45	1,973.93 ± 12.83	*p* < 0.01**	0.28
cerebellum total volume	103.12 ± 10.78	100.16 ± 11.25	0.01*	1.00
cerebellum rx	51.20 ± 5.35	49.75 ± 5.70	0.02*	1.00
cerebellum lx	51.92 ± 5.54	50.41 ± 5.67	0.01*	1.00
cerebellum asymmetry index ‡	−0.69 ± 1.54	−0.66 ± 1.58	0.86	1.00
insula total volume	18.52 ± 2.00	17.40 ± 1.93	*p* < 0.001***	*p* < 0.001***
insula rx	9.06 ± 0.99	8.49 ± 0.96	*p* < 0.001***	*p* < 0.001***
insula lx	9.46 ± 1.05	8.91 ± 1.03	*p* < 0.001***	*p* < 0.001***
insula asymmetry index	−2.12 ± 2.30	−2.38 ± 2.68	0.34	1.00
cingulate cortex total volume	31.15 ± 2.92	29.10 ± 2.91	*p* < 0.001***	*p* < 0.001***
cingulate cortex rx	15.65 ± 1.50	14.71 ± 1.60	*p* < 0.001***	*p* < 0.001***
cingulate cortex lx	15.50 ± 1.56	14.39 ± 1.44	*p* < 0.001***	*p* < 0.001***
cingulate cortex asymmetry index	0.48 ± 2.91	1.04 ± 3.26	0.10	1.00
hippocampus total volume ‡	8.95 ± 1.45	7.30 ± 1.35	*p* < 0.001***	*p* < 0.001***
hippocampus rx	4.36 ± 0.74	3.53 ± 0.72	*p* < 0.001***	*p* < 0.001***
hippocampus lx ‡	4.59 ± 0.76	3.77 ± 0.69	*p* < 0.001***	*p* < 0.001***
hippocampus asymmetry index ‡	−2.60 ± 4.34	−3.58 ± 6.29	0.14	1.00
parahippocampus total volume	8.60 ± 0.96	7.52 ± 0.93	*p* < 0.001***	*p* < 0.001***
parahippocampus rx ‡	4.66 ± 0.55	4.05 ± 0.56	*p* < 0.001***	*p* < 0.001***
parahippocampus lx ‡	3.94 ± 0.48	3.46 ± 0.47	*p* < 0.001***	*p* < 0.001***
parahippocampus asymmetry index ‡	8.47 ± 4.36	7.78 ± 5.74	0.25	1.00
amygdala total volume	2.72 ± 0.34	2.32 ± 0.36	*p* < 0.001***	*p* < 0.001***
amygdala rx	1.34 ± 0.16	1.14 ± 0.19	*p* < 0.001***	*p* < 0.001***
amygdala lx	1.38 ± 0.19	1.18 ± 0.20	*p* < 0.001***	*p* < 0.001***
amygdala asymmetry index ‡	−1.57 ± 4.58	−1.29 ± 6.26	0.59	1.00
ventral striatum total volume	1.84 ± 0.22	1.74 ± 0.22	*p* < 0.001***	*p* < 0.01**
ventral striatum rx	0.88 ± 0.11	0.82 ± 0.11	*p* < 0.001***	*p* < 0.01**
ventral striatum lx	0.97 ± 0.12	0.92 ± 0.12	*p* < 0.001***	*p* < 0.01**
ventral striatum asymmetry index ‡	−4.99 ± 2.93	−5.39 ± 4.17	0.35	1.00
thalamus total volume	10.39 ± 1.72	9.75 ± 1.75	*p* < 0.001***	0.06
thalamus rx ‡	5.39 ± 0.94	5.06 ± 0.96	*p* < 0.01**	0.15
thalamus lx	5.01 ± 0.81	4.69 ± 0.82	*p* < 0.001***	0.03*
thalamus asymmetry index ‡	3.57 ± 3.40	3.66 ± 4.09	0.37	1.00
precuneus total volume	23.86 ± 2.01	21.88 ± 2.25	*p* < 0.001***	*p* < 0.001***
precuneus rx	11.77 ± 1.05	10.83 ± 1.19	*p* < 0.001***	*p* < 0.001***
precuneus lx	12.10 ± 1.10	11.05 ± 1.21	*p* < 0.001***	*p* < 0.001***
precuneus asymmetry index	−1.37 ± 3.12	−1.02 ± 3.88	0.36	1.00
ft cortex total volume	292.06 ± 21.43	273.64 ± 22.89	*p* < 0.001***	*p* < 0.001***
ft cortex rx	146.95 ± 10.88	137.76 ± 11.87	*p* < 0.001***	*p* < 0.001***
ft cortex lx	145.11 ± 10.81	135.88 ± 11.70	*p* < 0.001***	*p* < 0.001***
ft cortex asymmetry index ‡	0.63 ± 1.16	0.68 ± 2.08	0.74	1.00
mt cortex total volume	17.55 ± 2.30	14.82 ± 2.19	*p* < 0.001***	*p* < 0.001***
mt cortex rx	9.03 ± 1.22	7.58 ± 1.23	*p* < 0.001***	*p* < 0.001***
mt cortex lx ‡	8.53 ± 1.16	7.24 ± 1.09	*p* < 0.001***	*p* < 0.001***
mt cortex asymmetry index ‡	2.84 ± 3.73	2.20 ± 5.51	0.28	1.00
po cortex total volume	126.43 ± 9.43	117.08 ± 11.28	*p* < 0.001***	*p* < 0.001***
po cortex rx	59.56 ± 4.46	55.30 ± 5.47	*p* < 0.001***	*p* < 0.001***
po cortex lx	66.86 ± 5.20	61.78 ± 6.24	*p* < 0.001***	*p* < 0.001***
po cortex asymmetry index ‡	−5.76 ± 1.65	−5.52 ± 2.77	0.14	1.00
Neuropsychological measures				
MMSE ‡	28.34 ± 1.59	25.38 ± 2.55	*p* < 0.001***	*p* < 0.001***
AVLT				
*Trial1 ‡*	4.89 ± 1.59	3.59 ± 1.41	*p* < 0.001***	*p* < 0.001***
*Trial1 errors ‡*	0.36 ± 0.74	0.49 ± 0.85	0.07	1.00
*Trial2 ‡*	6.94 ± 2.24	4.70 ± 1.49	*p* < 0.001***	*p* < 0.001***
*Trial2 errors ‡*	0.39 ± 0.78	0.50 ± 0.92	0.14	1.00
*Trial3 ‡*	8.38 ± 2.63	5.51 ± 1.83	*p* < 0.001***	*p* < 0.001***
*Trial3 errors ‡*	0.53 ± 0.90	0.51 ± 0.80	0.79	1.00
*Trial4 ‡*	9.29 ± 2.72	5.71 ± 1.92	*p* < 0.001***	*p* < 0.001***
*Trial4 errors ‡*	0.41 ± 0.96	0.53 ± 0.83	0.03*	1.00
*Trial5 ‡*	9.98 ± 2.86	5.91 ± 2.14	*p* < 0.001***	*p* < 0.001***
*Trial5 errors ‡*	0.25 ± 0.61	0.44 ± 0.78	0.01*	0.76
*Trial6 ‡*	6.87 ± 3.73	2.32 ± 2.09	*p* < 0.001***	*p* < 0.001***
*Trial6 errors ‡*	0.93 ± 1.31	1.08 ± 1.35	0.22	1.00
*Delayed ‡*	5.96 ± 4.02	1.33 ± 2.10	*p* < 0.001***	*p* < 0.001***
*Delayed errors ‡*	1.49 ± 1.63	1.27 ± 1.83	0.03*	1.00
*Recognitions ‡*	12.12 ± 2.89	8.09 ± 3.91	*p* < 0.001***	*p* < 0.001***
*Recognitions errors ‡*	1.34 ± 1.85	2.31 ± 2.32	*p* < 0.001***	*p* < 0.001***
*Digit Span Forward ‡*	6.67 ± 1.09	6.39 ± 1.05	0.02*	1.00
*Digit Span Backward ‡*	4.89 ± 1.22	4.28 ± 1.09	*p* < 0.001***	*p* < 0.001***
TMT-A				
*Time taken ‡*	34.71 ± 11.79	56.31 ± 28.66	*p* < 0.001***	*p* < 0.001***
*Committed errors ‡*	0.09 ± 0.43	0.22 ± 0.68	0.02*	0.97
*Omission errors ‡*	0.03 ± 0.38	0.05 ± 0.31	0.09	1.00
TMT-B				
*Time taken ‡*	85.95 ± 32.49	182.77 ± 83.80	*p* < 0.001***	*p* < 0.001***
*Committed errors ‡*	0.44 ± 0.68	1.62 ± 2.37	*p* < 0.001***	*p* < 0.001***
*Omission errors ‡*	0.10 ± 0.42	2.09 ± 4.86	*p* < 0.001***	*p* < 0.001***
Clock				
*Contour ‡*	0.99 ± 0.08	0.98 ± 0.13	0.30	1.00
*Number order ‡*	0.86 ± 0.35	0.63 ± 0.48	*p* < 0.001***	*p* < 0.001***
*Numbers present ‡*	0.93 ± 0.25	0.78 ± 0.41	*p* < 0.001***	*p* < 0.01**
*Hands ‡*	0.99 ± 0.11	0.87 ± 0.33	*p* < 0.001***	*p* < 0.01**
*Time signed ‡*	0.86 ± 0.35	0.48 ± 0.50	*p* < 0.001***	*p* < 0.001***
*Total score ‡*	4.64 ± 0.75	3.75 ± 1.15	*p* < 0.001***	*p* < 0.001***
Symbol digit	45.76 ± 9.42	30.08 ± 10.50	*p* < 0.001***	*p* < 0.001***
Category fluency				
*Animals ‡*	19.27 ± 5.38	14.12 ± 4.55	*p* < 0.001***	*p* < 0.001***
*Animals perseveration ‡*	0.84 ± 1.23	1.29 ± 1.68	0.02*	1.00
*Animals intrusion ‡*	0.06 ± 0.28	0.11 ± 0.67	0.89	1.00
*Vegetables ‡*	14.01 ± 4.13	9.22 ± 3.33	*p* < 0.001***	*p* < 0.001***
*Vegetables perseveration ‡*	0.52 ± 0.80	0.55 ± 0.93	0.98	1.00
*Vegetables intrusion ‡*	0.39 ± 1.00	0.92 ± 1.84	*p* < 0.01**	0.07
Boston Naming Test				
*Correct spontaneous answers ‡*	27.27 ± 2.94	23.63 ± 4.92	*p* < 0.001***	*p* < 0.001***
*Semantic cues ‡*	1.66 ± 2.62	3.48 ± 4.35	*p* < 0.001***	*p* < 0.01**
*Correct answer after semantic cue ‡*	0.30 ± 0.76	0.53 ± 1.13	0.04*	1.00
*Phonological cues ‡*	2.32 ± 2.69	5.58 ± 4.80	*p* < 0.001***	*p* < 0.001***
*Correct answer after phonological cue ‡*	0.30 ± 0.76	0.53 ± 1.13	0.04*	1.00
*Total score ‡*	27.57 ± 2.72	24.16 ± 4.79	*p* < 0.001***	*p* < 0.001***
FAQ				
*Finances ‡*	0.33 ± 0.91	2.44 ± 1.82	*p* < 0.001***	*p* < 0.001***
*Bills ‡*	0.41 ± 0.99	2.71 ± 1.86	*p* < 0.001***	*p* < 0.001***
*Buying ‡*	0.16 ± 0.62	1.84 ± 1.84	*p* < 0.001***	*p* < 0.001***
*Social life ‡*	0.21 ± 0.73	1.53 ± 1.65	*p* < 0.001***	*p* < 0.001***
*Housekeeping ‡*	0.02 ± 0.23	0.71 ± 1.44	*p* < 0.001***	*p* < 0.001***
*Cooking ‡*	0.18 ± 0.53	1.63 ± 1.63	*p* < 0.001***	*p* < 0.001***
*Keeping up with external events ‡*	0.12 ± 0.60	1.78 ± 1.83	*p* < 0.001***	*p* < 0.001***
*Entertainment and learning ‡*	0.05 ± 0.39	1.53 ± 1.66	*p* < 0.001***	*p* < 0.001***
*Memory ‡*	0.60 ± 1.30	3.07 ± 1.58	*p* < 0.001***	*p* < 0.001***
*Transports ‡*	0.07 ± 0.49	2.18 ± 2.10	*p* < 0.001***	*p* < 0.001***
*Total score ‡*	0.75 ± 1.72	9.02 ± 6.72	*p* < 0.001***	*p* < 0.001***

☨Bonferroni correction.

‡Non-parametric test.

SD, Standard deviation; rx, right; lx, left; CSF, cerebrospinal fluid; tiv, total intracranial volume; ft, fronto-temporal: frontal lobe + temporal lobe + anterior cingulate cortex; mt, mediotemporal; po, parieto-occipital: parietal lobe + occipital lobe; MMSE, Mini-Mental State Examination; AVLT, Auditory Verbal Learning Test; TMT, Trail Making Test; FAQ, Functional Assessment Questionnaire.

* for 0.01 < *p*-value < 0.05, ** for *p*-value < 0.01, *** for *p*-value < 0.001.

**Table 14:** Descriptive analysis of CSF biomarkers according to the AI-tool predicted risk of conversion or not to AD-dementia within 24-month using MRI and neuropsychological data (Subgroup V: *N =* 130).

	Low risk (*N =* 44)	High risk (*N =* 86)		
CSF biomarkers	Mean ± SD	Mean ± SD	*p*-value	*p*-value☨
Aβ42‡	899.85 ± 473.11	678.53 ± 362.78	0.01*	0.05
t-tau ‡	283.08 ± 91.80	366.08 ± 135.96	*p* < 0.001***	*p* < 0.01**
p-tau ‡	27.73 ± 10.73	36.85 ± 15.90	*p* < 0.01**	*p* < 0.01**
t-tau/Aβ42 ‡	0.42 ± 0.26	0.62 ± 0.29	*p* < 0.001***	*p* < 0.001***

☨Bonferroni correction.

‡Non-parametric test.

SD, Standard deviation; CSF, cerebrospinal fluid; Aβ42, amyloid-beta42; t-tau, total-tau; p-tau, phosphorylated-tau.

* for 0.01 < *p*-value < 0.05, ** for *p*-value < 0.01, *** for *p*-value < 0.001.

Spearman’s correlation results between brain-volumetric features and CSF proteins, calculated on the 482 participants with all CSF protein data, are presented in Table 15.

**Table 15:** Spearman’s correlation between brain-volumetric features and CSF proteins (Aβ42, t-tau, and p-tau) Subgroup III (*N =* 482).

	Aβ42	t-tau	p-tau
whole brain total volume	0.28	−0.18	−0.2
	*p* < 0.001***	*p* < 0.001***	*p* < 0.001***
whole brain perc over tiv	0.28	−0.18	−0.2
	*p* < 0.001***	*p* < 0.001***	*p* < 0.001***
whole brain rx	0.28	−0.18	−0.2
	*p* < 0.001***	*p* < 0.001***	*p* < 0.001***
whole brain lx	0.28	−0.19	−0.2
	*p* < 0.001***	*p* < 0.001***	*p* < 0.001***
whole brain asymmetry index	−2.90E-03	0.01	0.01
	0.95	0.76	0.78
gray matter total volume	0.32	−0.17	−0.19
	*p* < 0.001***	*p* < 0.001***	*p* < 0.001***
gray matter perc over tiv	0.32	−0.17	−0.19
	*p* < 0.001***	*p* < 0.001***	*p* < 0.001***
gray matter rx	0.32	−0.16	−0.19
	*p* < 0.001***	*p* < 0.001***	*p* < 0.001***
gray matter lx	0.32	−0.17	−0.19
	*p* < 0.001***	*p* < 0.001***	*p* < 0.001***
gray matter asymmetry index	0.03	−3.49E-03	−0.01
	0.54	0.94	0.79
white matter total volume	0.13	−0.12	−0.12
	*p* < 0.01**	*p* < 0.01**	0.01*
white matter perc over tiv	0.12	−0.12	−0.11
	*p* < 0.01**	*p* < 0.01**	0.01*
white matter rx	0.14	−0.12	−0.12
	*p* < 0.01**	*p* < 0.01**	0.01*
white matter lx	0.12	−0.12	−0.11
	0.01*	0.01*	0.01*
white matter asymmetry index	−0.07	−0.02	6.32E-04
	0.15	0.73	0.99
csf total volume	−0.28	0.18	0.2
	*p* < 0.001***	*p* < 0.001***	*p* < 0.001***
csf perc over tiv	−0.28	0.18	0.2
	*p* < 0.001***	*p* < 0.001***	*p* < 0.001***
tiv total volume	0.24	−0.09	−0.1
	*p* < 0.001***	0.06	0.03*
frontal lobe total volume	0.26	−0.1	−0.12
	*p* < 0.001***	0.03*	*p* < 0.01**
frontal lobe rx	0.25	−0.09	−0.11
	*p* < 0.001***	0.06	0.01*
frontal lobe lx	0.27	−0.1	−0.13
	*p* < 0.001***	0.02*	*p* < 0.01**
frontal lobe asymmetry index	−0.1	0.04	0.05
	0.02*	0.35	0.3
temporal lobe total volume	0.38	−0.22	−0.25
	*p* < 0.001***	*p* < 0.001***	*p* < 0.001***
temporal lobe rx	0.37	−0.21	−0.24
	*p* < 0.001***	*p* < 0.001***	*p* < 0.001***
temporal lobe lx	0.36	−0.22	−0.24
	*p* < 0.001***	*p* < 0.001***	*p* < 0.001***
temporal lobe asymmetry index	5.51E-04	−0.03	−0.01
	0.99	0.58	0.75
parietal lobe total volume	0.31	−0.19	−0.22
	*p* < 0.001***	*p* < 0.001***	*p* < 0.001***
parietal lobe rx	0.3	−0.18	−0.22
	*p* < 0.001***	*p* < 0.001***	*p* < 0.001***
parietal lobe lx	0.3	−0.18	−0.2
	*p* < 0.001***	*p* < 0.001***	*p* < 0.001***
parietal lobe asymmetry index	0.01	−0.04	−0.04
	0.75	0.4	0.34
occipital lobe total volume	0.28	−0.18	−0.2
	*p* < 0.001***	*p* < 0.001***	*p* < 0.001***
occipital lobe rx	0.28	−0.15	−0.17
	*p* < 0.001***	*p* < 0.001***	*p* < 0.001***
occipital lobe lx	0.27	−0.2	−0.21
	*p* < 0.001***	*p* < 0.001***	*p* < 0.001***
occipital lobe asymmetry index	0.02	0.07	0.06
	0.69	0.15	0.16
cerebellum total volume	0.13	−0.04	−0.04
	*p* < 0.01**	0.42	0.34
cerebellum rx	0.13	−0.03	−0.04
	*p* < 0.01**	0.48	0.41
cerebellum lx	0.14	−0.04	−0.05
	*p* < 0.01**	0.34	0.25
cerebellum asymmetry index	−0.06	0.07	0.08
	0.19	0.12	0.08
insula total volume	0.2	−0.04	−0.06
	*p* < 0.001***	0.39	0.2
insula rx	0.19	−0.04	−0.06
	*p* < 0.001***	0.39	0.21
insula lx	0.21	−0.03	−0.05
	*p* < 0.001***	0.46	0.24
insula asymmetry index	−0.02	−0.02	−0.02
	0.63	0.59	0.72
cingulate cortex total volume	0.26	−0.15	−0.17
	*p* < 0.001***	*p* < 0.001***	*p* < 0.001***
cingulate cortex rx	0.25	−0.12	−0.14
	*p* < 0.001***	*p* < 0.01**	*p* < 0.01**
cingulate cortex lx	0.26	−0.17	−0.18
	*p* < 0.001***	*p* < 0.001***	*p* < 0.001***
cingulate cortex asymmetry index	−0.02	0.08	0.08
	0.62	0.07	0.07
hippocampus total volume	0.41	−0.3	−0.32
	*p* < 0.001***	*p* < 0.001***	*p* < 0.001***
hippocampus rx	0.4	−0.3	−0.32
	*p* < 0.001***	*p* < 0.001***	*p* < 0.001***
hippocampus lx	0.4	−0.28	−0.3
	*p* < 0.001***	*p* < 0.001***	*p* < 0.001***
hippocampus asymmetry index	6.63E-03	−0.06	−0.05
	0.88	0.19	0.27
parahippocampus total volume	0.37	−0.29	−0.31
	*p* < 0.001***	*p* < 0.001***	*p* < 0.001***
parahippocampus rx	0.37	−0.3	−0.31
	*p* < 0.001***	*p* < 0.001***	*p* < 0.001***
parahippocampus lx	0.35	−0.26	−0.28
	*p* < 0.001***	*p* < 0.001***	*p* < 0.001***
parahippocampus asymmetry index	0.04	−0.08	−0.08
	0.37	0.06	0.08
amygdala total volume	0.41	−0.32	−0.33
	*p* < 0.001***	*p* < 0.001***	*p* < 0.001***
amygdala rx	0.39	−0.31	−0.33
	*p* < 0.001***	*p* < 0.001***	*p* < 0.001***
amygdala lx	0.39	−0.29	−0.31
	*p* < 0.001***	*p* < 0.001***	*p* < 0.001***
amygdala asymmetry index	−0.08	0.04	0.05
	0.08	0.38	0.31
ventral striatum total volume	0.21	−0.07	−0.09
	*p* < 0.001***	0.11	0.06
ventral striatum rx	0.2	−0.09	−0.1
	*p* < 0.001***	0.06	0.03*
ventral striatum lx	0.19	−0.06	−0.07
	*p* < 0.001***	0.22	0.12
ventral striatum asymmetry index	0.06	−0.05	−0.04
	0.22	0.28	0.32
thalamus total volume	0.23	−0.04	−0.07
	*p* < 0.001***	0.33	0.12
thalamus rx	0.23	−0.05	−0.08
	*p* < 0.001***	0.25	0.09
thalamus lx	0.23	−0.03	−0.06
	*p* < 0.001***	0.49	0.2
thalamus asymmetry index	8.62E-03	−0.08	−0.07
	0.85	0.1	0.14
precuneus total volume	0.31	−0.24	−0.27
	*p* < 0.001***	*p* < 0.001***	*p* < 0.001***
precuneus rx	0.3	−0.23	−0.26
	*p* < 0.001***	*p* < 0.001***	*p* < 0.001***
precuneus lx	0.28	−0.23	−0.26
	*p* < 0.001***	*p* < 0.001***	*p* < 0.001***
precuneus asymmetry index	0.03	−3.04E-03	−0.01
	0.45	0.95	0.79
ft cortex total volume	0.32	−0.16	−0.19
	*p* < 0.001***	*p* < 0.001***	*p* < 0.001***
ft cortex rx	0.31	−0.15	−0.17
	*p* < 0.001***	*p* < 0.01**	*p* < 0.001***
ft cortex lx	0.32	−0.16	−0.19
	*p* < 0.001***	*p* < 0.001***	*p* < 0.001***
ft cortex asymmetry index	−0.06	7.83E-03	0.02
	0.16	0.86	0.71
mt cortex total volume	0.41	−0.3	−0.33
	*p* < 0.001***	*p* < 0.001***	*p* < 0.001***
mt cortex rx	0.4	−0.31	−0.33
	*p* < 0.001***	*p* < 0.001***	*p* < 0.001***
mt cortex lx	0.4	−0.28	−0.31
	*p* < 0.001***	*p* < 0.001***	*p* < 0.001***
mt cortex asymmetry index	0.01	−0.08	−0.07
	0.77	0.09	0.14
po cortex total volume	0.32	−0.2	−0.23
	*p* < 0.001***	*p* < 0.001***	*p* < 0.001***
po cortex rx	0.31	−0.19	−0.22
	*p* < 0.001***	*p* < 0.001***	*p* < 0.001***
po cortex lx	0.32	−0.2	−0.23
	*p* < 0.001***	*p* < 0.001***	*p* < 0.001***
po cortex asymmetry index	−0.01	0.03	0.02
	0.78	0.49	0.59

rx, right; lx, left; CSF, cerebrospinal fluid; tiv, total intracranial volume; ft, fronto-temporal; mt, mediotemporal; po, posterior-occipital; Aβ42, amyloid-beta 42; t-tau, total-tau; p-tau, phosphorylated-tau. * for 0.01 < *p*-value < 0.05, ** for *p*-value < 0.01, *** for *p*-value < 0.001.

Spearman’s pairwise correlation results between brain-volumetric features and cognitive measures are presented in Supplementary Table 1.

## 4 Discussion

In this work, the performance of an AI tool applied to neuropsychological/neuroimaging assessment for supporting the staging, clinical profiling, diagnosis, causal hypothesis and progression of subjects at risk of AD, following Intersocietal recommendations, was assessed for a large population of subjects at risk of AD (795 subjects at risk of AD from 66 centers in US/Canada/Italy).

Patients performed neuropsychological tests, 3D MRI brain studies, CSF and PET studies. The cognitive and brain-volumetric features automatically processed by the AI tool were used to detect regional atrophy in specific brain regions and cognitive impairments in specific domains when compared with normative percentiles/cut-offs.

Performance of the AI tool were evaluated in: (1) classifying subjects as HS/SCI/WW, MCI/MD, and MSD, with respect to clinical staging performed by clinicians at baseline and at 24-month follow-up; (2) clinical profiling subjects, with respect to biomarker-based diagnosis for each stage; (3) predicting, at baseline, the conversion to AD-dementia within 24-month, with respect to clinical diagnosis at 24-month follow-up.

AI had a staging performance similar to that of clinicians in staging (Table 2). Inter-rater agreement (Cohen’s *k*) between AI and clinicians was substantial for both MCI/MD-vs-all (0.70) and HS/SCI/WW-vs-all (0.81) classification, almost perfect for MSD-vs-all (0.90) classification. However, 42% (47/112) HS/SCI/WW cases were restaged by AI as MCI and about ⅓ were AD based on CSF biomarkers. This was due to the more sensitive neuropsychological tests used by the AI for cognitive impairment assessment included in the battery of seven tests (see Section 2.2.1), not performed in baseline neurological visits. A more sensitive staging (more MCI detection for subjects with positive biomarkers) allows an earlier diagnosis and intervention with disease-modifying drugs for AD patients.

AI performance in causal hypothesis vs. biomarker-based diagnosis was 91% [84–96%95 CI] (positive predictive value), 100% [43.0–85.4%95 CI] (negative predictive value), and 91% [84–96%95 CI] (accuracy) (Table 4).

AI performance in predicting conversion to AD-dementia vs. clinical conversion to AD-dementia at 24-month follow-up was 89% [84–94%95 CI] (sensitivity), 82% [77–87%95 CI] (specificity), 85% [81–89%95 CI] (accuracy), 83% [79–87%95 CI] (ROC-AUC) (Tables 5, 6). This performance supports clinical profiling, clinical diagnosis and causal hypothesis and the optimal choice of first-line recommended biomarkers. To be noted, the AI tool was able to reduce the class of “no clear hypothesis” by the provision of the LR/HR to progress to AD-dementia within 24-month. However, a limitation of the study is the lack of subjects with Lewy body spectrum, motor tauopathy, or vascular dementia since these subjects were excluded by inclusion criteria during enrollment. This limitation can have an impact on the performance when the AI tool is used for clinical profiling of these clinical syndromes.

As expected, cognitive features decrease from MCI/MD to MSD (Table 10): major decreases occur in AVLT test (in the number of words recalled at stage 5, the last recall), in TMT-B test (in time taken for the task, in the number of omissions and committed errors); in Symbol Digit test (in the total score), in the test of Category fluency vegetables (in the number of vegetables), in FAQ (in activities related to finance and transportation). Consistently, brain-volumetric features, cognitive features and biomarkers change with subjects’ stage (Table 11). Brain-volumetric features decrease of about 3–10%: about 4% in WB, tiv, LX, RX, about 8% in medio-temporal cortex, about 6% in frontotemporal-cortex and in parieto-occipital cortex, and 10% in hippocampus. Consistently, CSF biomarkers decrease although not statistically significantly (Table 12).

Similarly, brain-volumetric, cognitive features and biomarkers change with subjects’ risk of conversion to AD-dementia (Tables 13, 14): all brain-volumetric features except for features representative of asymmetries and the thalamus and the cerebellum, as well as most cognitive features are significantly different. All biomarkers, except for Aβ42 are significantly different between the two groups (Table 14).

To be noted, all brain-volumetric features, except those representing asymmetries, are statistically significantly correlated with CSF biomarkers (Table 15). Moreover, most cognitive features are statistically significantly correlated with the brain-volumetric features (Supplementary Table 1), in particular: losses in AVLT and DIGIT SPAN FORWARD scores, most CLOCK scores and DIGIT SYMBOL total score are directly correlated with atrophy of the medio-temporal cortex and hippocampus. TMT-A time taken and TMT-B time taken/committed errors are inversely correlated with atrophy of the medio-temporal cortex and hippocampus. Interestingly, the number of animals/vegetables is directly correlated with the medio-temporal cortex and hippocampus and the number of animal perseverations is inversely correlated with the medio-temporal cortex and hippocampus. BNT total score and spontaneous answers are directly correlated with the medio-temporal cortex and hippocampus, while the phonological cues were inversely correlated with the medio-temporal cortex and hippocampus. Consistently, all FAQ subscores were inversely correlated with the medio-temporal cortex and hippocampus.

Previous studies have demonstrated the applicability of AI systems in analyzing MRI-T1 brain features and cognitive measures for supporting early diagnosis of AD and predicting subject-related risk of AD-dementia.

Among these studies, there are some that were conducted by researchers to support the safe design of software to be used as medical devices, based on SVM automatic classifiers using, as input, MRI-T1 brain features, eventually combined with cognitive measures of the subjects at risk of AD-dementia. In particular, we found the following studies reported in (65–67), that support the architectural choice of: (1) the image pre-processing method; (2) the feature extraction and selection method; (3) the classification metrics and validation procedures; (4) the output maps; (5) the ensemble of classifiers; and (6) the classification voting scheme.

Salvatore et al. (65) gave a state-of-the-art overview about the applicability of SVM automatic classifiers for the early and differential diagnosis of AD-related pathologies by means of MRI-T1 features, starting from preliminary steps such as image pre-processing, feature extraction, feature selection and ending with classification, validation strategies and extraction of MRI-related biomarkers. This study aims to provide a systematic overview about the SVM architecture in the automatic classification of AD subjects and in the prediction of conversion from MCI to AD-dementia. Both main achievements in terms of classification performance (e.g., accuracy, specificity and sensitivity) and limitations are described, including: (1) the effects of pre-processing on classification performances; (2) the effects of feature extraction and selection methods; (3) the effects of classification and validation procedure; (4) the interpretation of maps showing the importance of each MRI image voxel for the classification. The study is important because it provides evidence of the safe design choices that the manufacturer implemented in TRACE4AD in (1) image pre-processing method; (2) feature extraction and selection method; (3) classification metrics and validation procedures; (4) output maps showing the importance of each MRI image voxel for the classification for high explainability and interpretability of results of processing. No safety concerns were reported in this study.

Nanni et al. (66) proposed an ensemble of SVM automatic classifiers for the early diagnosis of AD similar to that developed by Salvatore et al. (25) based on different MRI-T1 features. The study reported results on testing the ensemble of SVM classifiers on different datasets of patients, including the same 509 ADNI patients tested in Salvatore et al. (25). Results showed that the proposed ensemble performs well in all the tested datasets. While the different feature selection approaches work differently in the different datasets, the proposed ensemble of SVM classifiers obtained good performance in all the datasets, allowing to prove high reliability. The study is important because it demonstrates the optimal choice of an architecture consisting of an ensemble of SVM classifiers for a reliable tool. No safety concerns were reported in this study.

Salvatore et al. (67), presented the results of the SVM automatic classifier for the analysis of MRI-T1 features, developed in the pivotal study of TRACE4AD published by Salvatore et al. (25), in the task of multi-label automatic classification of subjects: HS, ncMCI, cMCI, and AD, being cMCI and ncMCI those MCI subjects progressing or not to AD-dementia, respectively. This classifier was based on the previously developed SVM classifier and was combined with multi-label decision functions optimized and tested on the Kaggle web platform within the international challenge “A Machine learning neuroimaging challenge for automated diagnosis of Mild Cognitive Impairment.” The number of subjects enrolled was 400 subjects from the ADNI cohort, including 100 HS, 100 MCI not converter to Alzheimer’s dementia (ncMCI), 100 MCI converter to Alzheimer’s dementia (cMCI), and 100 AD. This 400-subjects dataset was then split into a training set and a testing set. The training set consisted of 240 subjects, while the testing set consisted of 160 subjects. The testing set was further inflated with 340 dummy subjects, reaching a total of 500 subjects in its final configuration. Results showed that the performance of multi-label automatic-classification systems strongly depends on the choice of the voting scheme used for combining binary-classification labels. Indeed, the voting scheme mainly based on the binary-classification performances on the different four groups is the best choice to model the multi-label decision function for AD, when compared with a simple majority-vote scheme or with a scheme aimed at discriminating patients with high vs. low risk of conversion to AD and therapy addressing. The accuracy of the SVM classifier was higher than or comparable to the previously published one. No safety concerns were reported in this study.

A study on a new automatic classification system for the early diagnosis and prognosis of AD was published by Nanni et al. (68) and is reported here since the system has many similar features with TRACE4AD. The study proposed a combination of texture descriptors with voxel-based features, extracted from the MRI-T1 study of the subjects’ brain, as input to an ensemble of SVM classifiers for the early diagnosis of AD. The authors compared the performance of their system with the performance of the SVM ensemble developed by Salvatore et al. in 2015. and found an improvement in the sensitivity performance, although specificity was <70%. In particular, on the sole binary comparison between “patients with AD or developing AD” (AD and cMCI) and “patients without AD or not-converter to Alzheimer’s dementia” (HS and ncMCI), thus excluding any further multi-label decision function, the proposed classification system was able to correctly predict the two groups of subjects with an accuracy of 77%, a sensitivity of 90%, and a specificity of 64%. No safety concerns were reported in this study. However, the tool is not registered in any medical device databases.

Relevant performance and clinical outcome parameters for the intended clinical benefits from the above-mentioned published clinical data were obtained from cohorts of patients on the order of a few hundred at risk of AD-dementia. Overall, the state of the art confirmed the safety and effective performance of SVM systems for the analysis of MRI-T1 brain features and cognitive measures and their positive impact on the clinical workflow in supporting physicians for the reporting, diagnosis and prognosis of patients at risk of AD-dementia.

The evaluation of other medical device software highlights the landscape of automated MRI volumetry tools used for AD and other neurodegenerative conditions. Similar medical devices available on the market have been identified in medical device databases sharing similar characteristics with TRACE4AD.

Icobrain (Icometrix) reports abundant clinical data in the scientific literature. The most important ones include clinical data on the validation and the diagnostic performance of the software, published by Struyfs et al. (62). In this study the authors describe and validate icobrain dm, an automatic tool that segments brain structures that are relevant for differential diagnosis of dementia, such as the hippocampi and cerebral lobes. When comparing volumes obtained from AD patients against age-matched HS, all measures achieved high diagnostic performance levels when discriminating patients from HS, with the temporal cortex volume measured by icobrain dm reaching the highest diagnostic performance level (area under the receiver operating characteristic curve = 0.99) in this dataset. Results on the diagnostic value of Icobrain are also published by Wittens et al. (69). This study examines the diagnostic value of icobrain dm for AD in routine clinical practice, including a comparison to the widely used FreeSurfer software, and investigates if combined brain volumes contribute to establishing an AD diagnosis. The study population included HS (*n* = 90), SCI (*n* = 93), MCI (MCI, *n* = 357), and AD-dementia (*n* = 280) patients. Through automated volumetric analyses of global, cortical, and subcortical brain structures on clinical brain MRI-T1w (*n* = 820) images from a retrospective, multi-center study [REMEMBER, (70)], icobrain dm’s (v.4.4.0) ability to differentiate disease stages via ROC analysis was compared to FreeSurfer (v.6.0). Stepwise backward regression models were constructed to investigate if combined brain volumes can differentiate between AD stages. Results show that icobrain dm outperformed FreeSurfer in processing time (15–30 min versus 9–32 h), robustness (0 versus 67 failures), and diagnostic performance for whole brain, hippocampal volumes, and lateral ventricles between HS and AD-dementia patients. Stepwise backward regression showed improved diagnostic accuracy for pairwise group differentiations, with the highest performance obtained for distinguishing HS from AD-dementia (AUC = 0.914; specificity 83.0%; sensitivity 86.3%). The authors concluded that the automated volumetry has a diagnostic value for AD diagnosis in routine clinical practice. Their findings indicate that combined brain volumes improve diagnostic accuracy, using real-world imaging data from a clinical setting.

Clinical data on the medical device software Quantib ND (Quantib, Rotterdam, the Netherlands; now part of DeepHealth) are published by Poos et al. (60). This study highlights the value of normative volumetry software for disease tracking and staging biomarkers in genetic fronto-temporal dementia (FTD) showing how these techniques can help in identifying the optimal time window for starting treatment and monitoring treatment response. More specifically, the study investigates longitudinal brain atrophy rates in the presymptomatic stage of genetic FTD using the normative brain volumetry software Quantib for brain structures. Presymptomatic GRN, MAPT, and C9orf72 pathogenic variant carriers underwent longitudinal volumetric MRI-T1w of the brain as part of a prospective cohort study. Images were automatically analyzed with Quantib ND, which consisted of volume measurements (CSF and sum of gray and white matter) of lobes, cerebellum, and hippocampus. All volumes were compared with reference centile curves based on a large population-derived sample of nondemented individuals. Mixed-effects models were fitted to analyze atrophy rates of the different gene groups as a function of age. Thirty-four GRN, 8 MAPT, and 14 C9orf72 pathogenic variant carriers were included (mean age = 52.1, standard deviation = 7.2; 66% female). The mean follow-up duration of the study was 64 ± 33 months (median = 52; range 13–108). GRN pathogenic variant carriers showed a faster decline than the reference centile curves for all brain areas, though relative volumes remained between the 5th and 75th percentiles between the ages of 45 and 70 years. In MAPT pathogenic variant carriers, frontal lobe volume was already at the 5th percentile at age 45 years and showed a further decline between the ages of 50 and 60 years. Temporal lobe volume started in the 50th percentile at age 45 years but showed a faster decline over time compared with other brain structures. Frontal, temporal, parietal, and cerebellar volume already started below the 5th percentile compared with the reference centile curves at age 45 years for C9orf72 pathogenic variant carriers, but there was minimal decline over time until the age of 60 years. Other clinical data have been reported and compared for the devices Quibim Precision Brain Atrophy Screening and Quantib ND by Zak et al. (71). The authors compared the two AI software packages performing normative brain volumetry and explored whether they could differently impact dementia diagnostics in a clinical context. Sixty patients (20 AD, 20 FTD, 20 MCI) and 20 HS were included retrospectively. One MRI per subject was processed by software packages from the two proprietary manufacturers, producing two quantitative reports per subject. Two neuroradiologists assigned forced-choice diagnoses using only the normative volumetry data in these reports. They classified the volumetric profile as “normal,” or “abnormal,” and if “abnormal,” they specified the most likely dementia subtype. Differences between the packages’ clinical impact were assessed by comparing (1) agreement between diagnoses based on software output; (2) diagnostic accuracy, sensitivity, and specificity; and (3) diagnostic confidence. Quantitative outputs were also compared to provide context to any diagnostic differences. Diagnostic agreement between packages was moderate, for distinguishing normal and abnormal volumetry (*K* = 0.41–0.43) and for specific diagnoses (*K* = 0.36–0.38). However, each package yielded high inter-observer agreement when distinguishing normal and abnormal profiles (*K* = 0.73–0.82). Accuracy, sensitivity, and specificity were not different between packages. Diagnostic confidence was different between packages for one rater. Whole brain intracranial volume output differed between software packages (10.73%, *p* < 0.001), and normative regional data interpreted for diagnosis correlated weakly to moderately (rs = 0.12–0.80). The authors concluded that different artificial intelligence software packages for quantitative normative assessment of brain MRI can produce distinct effects at the level of clinical interpretation and that clinics should not assume that different packages are interchangeable, thus recommending internal evaluation of packages before adoption.

Based on the features and performance reported in this study, TRACE4AD can play a significant role in the evolving landscape of AD diagnosis and treatment, particularly when combined with emerging disease-modifying therapies. From an individual-patient perspective, TRACE4AD facilitates the identification of MCI likely to progress to AD within 2 years. This targeted approach can enable a more efficient evaluation of therapeutic effects over different time frames, in particular at intervals of 24-months, ultimately increasing the power to detect cognitive impairment progression.

From a different perspective, by identifying individuals likely to convert to dementia within 24-months, TRACE4AD helps shorten clinical trials. The tool aids in evaluating the effects of treatment when selecting MCI patients at higher risk of progressing to AD within 2 years, rather than those with a more stable cognitive condition, during the screening process for eligibility assessment in clinical trials and in stratifying AD subjects into rapid and slow progressors. This approach can ultimately reduce trial-associated costs, and address challenges related to high screen failure rates and the inclusion of heterogeneous participants in patients’ groups (72).

We highlight that a longer follow-up period would offer more comprehensive results into the tool’s ability to predict longer-term outcomes. Moreover, although beyond the scope of the present study, the presence of more subjects with non-AD dementia types in TRACE4AD analysis (e.g., FTD, motor tauopathy and Lewy body dementia) would add more findings.

Even though the present study and the current state-of-the-art literature have proven the usefulness of AI in neuroimaging, several ethical challenges should be taken into account. AI should support clinicians in their decision-making process, not favoring job displacement but promoting the powerful cooperation between AI and healthcare professionals. In a clinical setting, this cooperation should be encouraged by an explainable AI model reasoning to clinicians and patients following transparency and accountability principles. A responsible implementation and use of AI tools must be ensured by the definition of data security and privacy measures (73). In the case of TRACE4AD, this is ensured by the manufacturer’s declared compliance of the development process with the latest and highest standards of safety and security for AI-based medical devices, including BS AAMI 34971 (46) and MDCG 2019–16 (48), as well as with European Regulation 2024/1689 (AI ACT) (49), European Regulations 2016/679 (50), 2018/1725 (51) and European Directive 2016/680 (52).

Moreover, AI models should be trained and validated on varied datasets to improve model generalizability, to guarantee biases/errors prevention (73) and to ensure the adherence to fairness principles (74). Although different ethnicities and racial categories were represented in the considered validation population, non-Hispanic or Latino patients accounted for 97.1% of the total population, while 2.6% were Hispanic or Latino (ethnicity of 0.4% could not be determined); with respect to racial category, the population consisted predominantly of white patients (81.4%), black or African American (4.1%), asian (2.6%), native Hawaiian or pacific islander (0.1%), while for 1.8% the racial categories were more than one and for 10% it could not be determined; socio-economic groups were determined according to the level of education, whose mean value across the entire set of patients was calculated to be 16.38 years with a standard deviation of 2.70.

An assessment of the cost-effectiveness of TRACE4AD is out of the purpose of the present study. However, the commercial tool with similar intended use and operational aspects of TRACE4AD in clinical settings, Icobrain (Icometrix), above mentioned, has published an independent assessment of cost-effectiveness on feasibility for widespread clinical adoption. The assessment showed that the health economic impact per patient per year in using such a tool is estimated as $1,500–$2,200 in cost savings (75). Based on these findings, the American Medical Association (AMA) has issued a Current Procedural Terminology (CPT®) code for the tool FDA-cleared, (“AI-related brain MRI quantification software”), thereby creating a path to reimbursement (codes 0865 T and 0866 T). Thus, in the US, Medicare, Medicaid, and commercial health plans use CPT® codes to identify healthcare procedures and services. Once in effect, hospitals and imaging centers can use the new CPT® codes to submit claims for Icometrix’s AI-based analysis of brain MRI scans. It is mentioned that quantitative imaging analysis reported by code 0866 T is used for patients with multiple sclerosis, AD, traumatic brain injury, stroke, epilepsy, and Parkinson’s disease. Subtle areas of abnormality that are not easily detected by the human eye are identified and compared with previous MR imaging to determine changes and disease progression. These cost-effectiveness assessments provide indirect information on the positive benefits that AI devices that support medical specialists in the process of assessing patients to reach an accurate AD diagnosis can have.

AI tools could benefit from including additional neuroimaging techniques (such as functional MRI or PET scans) to compare the efficacy of the AI tool across different types of brain imaging (64, 76). However, it must be underlined that the aim of the present study was to assess the support of AI to automatically process structural MRI brain studies combined with neuropsychological scores, as required by the Intersocietal recommendations for all patients in Wave 1, irrespectively from the suspected diagnosis (15); PET is recommended only in Wave 2 for a suspected FTLD or motor tauopathy, as alternative to CSF biomarkers for a suspected diagnosis of AD; functional MRI is not recommended in the proposed clinical brain imaging protocol proposed by the societies’ consensus.

In conclusion, the performance of an AI tool was assessed when applied to the neuropsychological/neuroimaging assessment of subjects at risk of AD, following recommendations from 11 European scientific societies/organizations and a patient advocacy association (Alzheimer’s Europe) for the optimal patient-centered biomarker-based diagnostic workflow in memory clinics.

The AI tool was proved effective in supporting staging, clinical profiling, diagnosis, causal hypothesis and progression (risk to convert) to AD-dementia within 24-month supporting clinical management of AD patients.

The tool is intended to be used by specialized clinicians, in particular in memory clinics, as a decision support system for a personalized early diagnosis, prognosis and intervention of patients at risk of AD.

## Group member of Alzheimer’s Disease Neuroimaging Initiative

Data used in preparation of this article were obtained from the Alzheimer’s Disease Neuroimaging Initiative (ADNI) database (adni.loni.usc.edu). As such, the investigators within the ADNI contributed to the design and implementation of ADNI and/or provided data but did not participate in analysis or writing of this report. A complete listing of ADNI investigators can be found at: http://adni.loni.usc.edu/wp-content/uploads/how_to_apply/ADNI_Acknowledgement_List.pdf.

## Data Availability

The datasets presented in this article are not readily available because a subset of the dataset is from private cohort of Italian hospitals. Requests to access the datasets should be directed to Isabella Castiglioni, isabella.castiglioni@unimib.it.
